# An annotated catalogue of the mayfly fauna of Turkey (Insecta, Ephemeroptera)

**DOI:** 10.3897/zookeys.620.9405

**Published:** 2016-09-29

**Authors:** Ali Salur, Mustafa Cemal Darilmaz, Ernst Bauernfeind

**Affiliations:** 1 Hitit University, Faculty of Arts and Science, Department of Biology, Çorum, Turkey; 2 Aksaray University, Faculty of Arts and Science, Department of Biology, 68100 Aksaray, Turkey; 3 Naturhistorisches Museum Wien, Vienna, Austria

**Keywords:** Annotated catalogue, bibliography, Ephemeroptera, Turkey

## Abstract

The mayfly fauna of Turkey was reviewed including all hitherto known distribution records together with references and a few new records. Additionally, comments on taxonomy, identification and nomenclature are provided. Two species are new for the Turkish fauna: *Ephemera romantzovi* Kluge, 1988 and *Thraulus thraker* Jacob, 1988. A list of taxa including their recorded distribution in Turkey (according to provinces) is provided in the annotated catalogue. The type locality is also given for each species originally described from Turkey. According to the literature and the new records, 157 mayfly taxa representing 33 genera and 14 families were described from Turkey. Among them, 24 species are considered endemic to Anatolia.

## Introduction

Turkey is located among three continents geographically and covers a region also known as Asia Minor and Anatolia. Ulmer (1920) was the first author who described a new mayfly taxon from Turkey, whereas Verrier (1955) and Puthz (1972) provided the first faunistic records. Puthz (1978) already listed 17 species from Turkey but earnest faunistic research commenced with Kazancı (1984)^1^, who contributed so far more than 30 papers, followed by Tanatmış (from 1995 onwards)^2^ and others. A total of more than 70 scientific papers and books have been published on Ephemeroptera in Turkey until the year 2015 by Turkish and foreign researchers.

The websites www.faunaturkey.com and www.faunaturkey.org (established in 2013) aim to contribute more information on researchs about the fauna of Turkey. The data provided will be also added to the websites after publication. Our hope is to keep this list up-to-date with further additions and some corrections periodically, so we welcome information on any omissions, errors, and updates.

## Material and methods

Data in this review have been based on a detailed study of literature on Ephemeroptera in Turkey as well as on hitherto unpublished material housed in the Natural History Museum, Vienna (NMW, Austria). Unpublished theses have not been considered, nor have all records above the species level. Distribution of species-group taxa in Turkey has been listed and referenced according to publication dates. National distribution records (without specific data at least on province level) have been listed under ‘Turkey’. Type locality of species were only provided if the taxon had originally been based on material from Turkey. Additionally, taxa considered endemic to Turkey have been specifically mentioned under ‘*note*’. Remarks on different taxonomic opinions and nomenclature have been added under ‘*Comment*’ whenever appropriate. Nomenclature and arrangement of families are given according to Bauernfeind and Soldán (2012).

**Figure 1.**
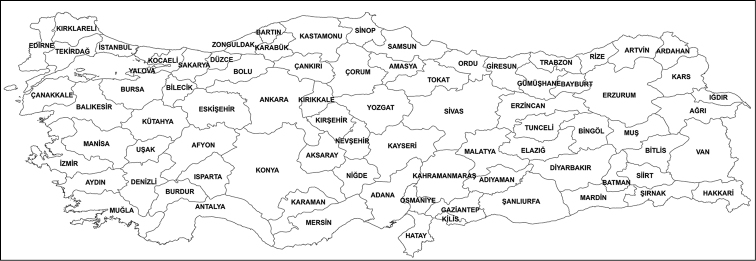
Provinces of Turkey

### Annotated catalogue of the Turkish mayfly fauna

157 species-group taxa (153 species and 4 subspecies) of mayflies representing 33 genera and 14 families have been recorded from Turkey. Among them, 24 species (15.3%) are presently considered endemic to Turkey. Three species have been excluded from the Turkish checklist.

### Family AMELETIDAE McCafferty, 1991

#### Genus *Ameletus* Eaton, 1885

***Ameletus inopinatus* Eaton, 1887**

**Distribution in Turkey.** Ankara, Eskişehir (Tanatmış 1995); Kırklareli, Tekirdağ (Tanatmış 1997); Samsun, Zonguldak (Kazancı 2001b); listed from Turkey: Tanatmış (1999); Kazancı and Türkmen (2012).

#### Genus *Metreletus* Demoulin, 1951

***Metreletus balcanicus* (Ulmer, 1920)**

**Distribution in Turkey.** Kırklareli (Kazancı 1998a); listed from Turkey: Kazancı (2001b); Kazancı and Türkmen (2012).

### Family SIPHLONURIDAE Ulmer, 1920

#### Genus *Siphlonurus* Eaton, 1868

***Siphlonurus aestivalis* Eaton, 1903**

**Distribution in Turkey.** Kırklareli, Tekirdağ (Tanatmış 1997); Balıkesir, Kütahya (Tanatmış 2000); Kütahya (Tanatmış 2002); Bolu (Tanatmış 2004a); Balıkesir (Narin and Tanatmış 2004); Konya (Kazancı 2011); listed from Turkey: Tanatmış (1999); Kazancı (2001b); Kazancı and Türkmen (2012).

**Comment.** Considering the difficulties in identification of *Siphlonurus* taxa at the larval stage, records based on male imagines would be desirable.

***Siphlonurus lacustris* Eaton, 1870**

**Distribution in Turkey.** Aydın (Kazancı 2001b); listed from Turkey: Kazancı and Türkmen (2012).

**Braasch, 1983**

**Type country and locality.** Turkey, approximately 170-180 km south-east Amasia, Reşadiye district, the province of Tokat (Braasch 1983a).

**Distribution in Turkey.** Tokat (Braasch 1983a); listed from Turkey: Tanatmış (1999); Kazancı and Türkmen (2012).

**Comment.** Larva not described.

**Note.** Endemic to Turkey.

### Family BAETIDAE Leach, 1815

#### Genus *Baetis* Leach, 1815

##### Subgenus Acentrella Bengtsson, 1912

**Comment.** *Acentrella* is either considered of generic rank (Barber-James et al. 2013) or a subgenus of *Baetis* (Novikova and Kluge 1987; Bauernfeind and Soldán 2011); see also Kluge and Novikova (2011).

**Baetis (Acentrella) inexpectatus (Tshernova, 1928)**

**Distribution in Turkey.** Elazığ (Berker 1981); Ağrı, Kars (Kazancı 1986a); listed from Turkey: Tanatmış (1999); Kazancı (2001b); Kazancı and Türkmen (2012).

**Baetis (Acentrella) lapponicus (Bengtsson, 1912)**

**Distribution in Turkey.** Bolu (Kazancı 2001b); Bolu (Kazancı and Türkmen 2008a); Bolu (Kazancı and Türkmen 2008b); listed from Turkey: Kazancı and Türkmen (2012).

**Comment.** Occurrence of Baetis (Acentrella) lapponicus in Turkey [syntopic with Baetis (Acentrella) sinaicus] seems rather doubtful and a re-examination of voucher specimens would be useful.

**Baetis (Acentrella) sinaicus (Bogoescu, 1931)**

**Distribution in Turkey.** Bolu (Kazancı 1984); Balıkesir (Tanatmış 2000); Karabük (Tanatmış 2004a); listed from Turkey: Tanatmış (1999); Kazancı (2001b); Kazancı and Türkmen (2012).

##### Subgenus Baetis Leach, 1815

**Baetis (Baetis) alpinus (Pictet, 1843)**

**Distribution in Turkey.** Ankara, Ağrı, Antalya, Bayburt, Erzurum, Kars, Konya (Kazancı 1984); Çankırı, Erzincan, Hakkari (Kazancı 2009); listed from Turkey: Puthz (1973); Tanatmış (1999); Kazancı (2001b); Kazancı and Türkmen (2012).

**Comment.** Taxonomy of the *Baetis alpinus* species-group *sensu* Müller-Liebenau (1969) is rather complicated and several taxa are known from the near vicinity of Turkey. Tabular summaries of diagnostic characters for all species of the *Baetis alpinus* species-group described so far have been provided by Peru and Thomas (2003, Ephemera 3, 2: 75) for larvae and by Righetti and Thomas (2001, Ephemera 2, 2: 77) for male imagines.

**Baetis (Baetis) buceratus Eaton, 1870**

**Distribution in Turkey.** Ankara, Antalya, Balıkesir, Bayburt, Bingöl, Bolu, Elazığ, Erzurum, Eskişehir, Isparta, Kırşehir, Konya, Sivas, Van (Kazancı 1985a); Hatay, Şanlıurfa (Koch 1988); Muğla (Kazancı et al. 1992); Balıkesir (Tanatmış 2000); Balıkesir, Bursa, Kütahya (Tanatmış 2002); Bolu, Karabük, Kastamonu, Zonguldak (Tanatmış 2004a); Kastamonu (Tanatmış 2004b); Balıkesir, Çanakkale (Narin and Tanatmış 2004); Sinop (Ertorun and Tanatmış 2004); Bartın (Tanatmış and Ertorun 2006); Düzce, Zonguldak (Tanatmış 2007); Ankara (Kazancı and Girgin 2008); Adıyaman, Erzurum, Kars (Kazancı 2009); Afyon, Konya (Özyurt and Tanatmış 2011); Malatya (Aydınlı 2013); İzmir, Kütahya, Manisa, Uşak (Aydınlı and Ertorun 2015); listed from Turkey: Tanatmış (1999); Kazancı (2001b); Kazancı and Türkmen (2012).

**Baetis (Baetis) elazigi Berker, 1981**

**Type country and locality.** Turkey, Keban Deresi (the type locality is located in the province of Elazığ) (Berker 1981).

**Distribution in Turkey.** Elazığ (Berker 1981); listed from Turkey: Tanatmış (1999); Kazancı (2001b); Kazancı and Türkmen (2012).

**Note.** Endemic to Turkey

**Comment.** Description and drawings do not allow identification without some doubt. A re-examination of type material (not specified) is necessary to ascertain the taxonomic status of this species.

**Baetis (Baetis) fuscatus (Linnaeus, 1761)**

**Distribution in Turkey.** Hatay [as *Baetis bioculatus* Linnaeus, 1758 (Verrier 1955)]; Ankara, Ağrı, Balıkesir, Bayburt, Bingöl, Erzurum, Erzincan, Hatay, Kars, Muş, Tekirdağ (Kazancı 1985a); Tekirdağ (Tanatmış 1997); Afyon (Kazancı 1998b); Balıkesir (Tanatmış 2000); Erzincan, Erzurum, Gümüşhane, Kars (Kazancı 2001a); Bursa, Kütahya (Tanatmış 2002); Bolu, Karabük, Zonguldak (Tanatmış 2004a); Kastamonu, Sinop (Tanatmış 2004b); Balıkesir, Çanakkale (Narin and Tanatmış 2004); Sinop (Ertorun and Tanatmış 2004); Bartın (Tanatmış and Ertorun 2006); Düzce, Zonguldak (Tanatmış 2007); Ankara (Kazancı and Girgin 2008); Bolu (Kazancı and Türkmen 2008a); Bolu (Kazancı and Türkmen 2008b); Giresun, Gümüşhane, Rize, Trabzon (Türkmen and Kazancı 2015); İzmir, Manisa (Aydınlı and Ertorun 2015); listed from Turkey: Tanatmış (1999); Kazancı (2001b); Kazancı and Türkmen (2012); Eastern Black Sea Basin (Türkmen and Kazancı 2013).

**Baetis (Baetis) lutheri Müller-Liebenau, 1967**

**Distribution in Turkey.** Muş (Kazancı 1985a); Sivas (Koch 1985); Hatay (Koch 1988); Muğla (Kazancı et al. 1992); Balıkesir (Tanatmış 2000); Ankara (Kazancı 2001b); Bursa (Tanatmış 2002); Bolu, Karabük, Kastamonu, Zonguldak (Tanatmış 2004a); Kastamonu, Sinop (Tanatmış 2004b); Balıkesir (Narin and Tanatmış 2004); Sinop (Ertorun and Tanatmış 2004); Bartın (Tanatmış and Ertorun 2006); Düzce (Tanatmış 2007); Ankara (Kazancı and Girgin 2008); Bolu (Kazancı and Türkmen 2008a); Bolu (Kazancı and Türkmen 2008b); Sinop (Tanatmış and Ertorun 2008); Adıyaman (Kazancı 2009); Afyon, Konya (Özyurt and Tanatmış 2011); Tokat (Kazancı et al. 2012); Malatya (Aydınlı 2013); Giresun, Gümüşhane, Rize, Trabzon (Türkmen and Kazancı 2015); Kütahya, Manisa, Uşak (Aydınlı and Ertorun 2015); listed from Turkey: Tanatmış (1999); Kazancı and Türkmen (2012); Eastern Black Sea Basin (Türkmen and Kazancı 2013).

**Comment.** Subspecific identity of records as *Baetis lutheri* (*as above*) is not clear.

**Baetis (Baetis) lutheri georgiensis Zimmermann, 1981**

**Distribution in Turkey.** Artvin, Erzincan, Tunceli (Kazancı 2009); listed from Turkey: Kazancı and Türkmen (2012).

**Comment.** Larval characters of *Baetis lutheri georgensis* Zimmermann have so far not been described. Identification and separation of subspecies *Baetis lutheri lutheri* Müller-Liebenau and *Baetis lutheri georgensis* Zimmermann in the larval stage remain therefore doubtful at present.

**Baetis (Baetis) macani Kimmins, 1957**

**Distribution in Turkey.** Elazığ (Berker 1981); listed from Turkey: Tanatmış (1999); Kazancı (2001b); Kazancı and Türkmen (2012).

**Comment.** Taxonomy and identification of *Baetis macani* and related taxa is rather complicated (see Savolainen et al. 2007; Savolainen 2009). *Baetis macani* is considered to represent a tundral or boreo-tundral faunistic element distributed north of 54° northern latitude and occurrence in Turkey is rather unlikely. A re-examination of voucher material would be useful.

**Baetis (Baetis) melanonyx (Pictet, 1843)**

**Distribution in Turkey.** Hatay (Koch 1988); Ankara (Kazancı 2001b); listed from Turkey: Kazancı and Türkmen (2012).

**Baetis (Baetis) meridionalis Ikonomov, 1954**

**Distribution in Turkey.** Ankara, Muş, Sivas (Kazancı 1984); Balıkesir, Bursa, Kütahya (Tanatmış 2000); Balıkesir, Bursa, Kütahya (Tanatmış 2002); listed from Turkey: Tanatmış (1999); Kazancı (2001b); Kazancı and Türkmen (2012).

**Baetis (Baetis) pavidus Grandi, 1951**

**Distribution in Turkey.** Elazığ (Berker 1981); Bilecik, Bursa, Eskişehir, Kütahya (Tanatmış 1995); Balıkesir, (Tanatmış 2000); Bursa, Kütahya (Tanatmış 2002); Bolu (Kazancı and Türkmen 2008a); Bolu (Kazancı and Türkmen 2008b); Tokat (Kazancı et al. 2012); listed from Turkey: Tanatmış (1999); Kazancı (2001b); Kazancı and Türkmen (2012).

**Baetis (Baetis) nexus Navás, 1918**

**Distribution in Turkey** (as *Baetis pentaphlebodes* Ujhelyi, 1966). Kars, Erzurum (Kazancı 1984); Kütahya (Tanatmış 2000, 2002); listed from Turkey: Tanatmış (1999); Kazancı (2001b); Kazancı and Türkmen (2012).

**Comment.** *Baetis pentaphlebodes* Ujhelyi, 1966 is usually considered to represent a junior subjective synonym of *Baetis nexus* Navás, 1918 (International Commission on Zoological Nomenclature 2007. Opinion 2171, Case 3322. Bulletin of Zoological Nomenclature 64 (2): 131 [*Baetis nexus* Navás, 1918 placed on the Official List of Specific Names in Zoology]. See also Sziráki (2005).

**Baetis (Baetis) samochai Koch, 1981**

**Distribution in Turkey.** Diyarbakır (Koch 1985); listed from Turkey: Tanatmış (1999); Kazancı (2001b); Kazancı and Türkmen (2012).

**Baetis (Baetis) scambus Eaton, 1870**

**Distribution in Turkey.** Ankara (Kazancı 1984); Muğla (Kazancı et al. 1992); Ankara, Bursa, Eskişehir, Kütahya (Tanatmış 1995); Bolu, Çankırı, Kırşehir (Kazancı 2001b); Bolu (Taşdemir et al. 2008); listed from Turkey: Tanatmış (1999); Kazancı and Türkmen (2012).

**Comment.** Larvae are very similar to *Baetis fuscatus*, not always reliably separated. Identification is comparatively easy if larvae and male imagines are associated.

**Baetis (Baetis) vardarensis caucasicus Zimmermann, 1981**

**Distribution in Turkey.** Trabzon (Kazancı 2009); listed from Turkey: Kazancı and Türkmen (2012).

**Baetis (Baetis) vernus Curtis, 1834**

**Distribution in Turkey.** Elazığ (Berker 1981); Ankara, Erzincan (Kazancı 1984); Sivas (Koch 1985); Ankara, Bolu, Eskişehir, Kütahya (Tanatmış 1995); Edirne, İstanbul, Kırklareli, Tekirdağ (Tanatmış 1997); Balıkesir, Bursa, Kütahya (Tanatmış 2000); Erzincan, Erzurum (Kazancı 2001a); Kırşehir, Konya (Kazancı 2001b); Bursa, Kütahya (Tanatmış 2002); Bolu, Karabük, Kastamonu, Zonguldak (Tanatmış 2004a); Kastamonu, Sinop (Tanatmış 2004b); Balıkesir (Narin and Tanatmış 2004); Sinop (Ertorun and Tanatmış 2004); Bartın (Tanatmış and Ertorun 2006); Düzce (Tanatmış 2007); Sinop (Tanatmış and Ertorun 2008); Afyon, Konya (Özyurt and Tanatmış 2011); Malatya (Aydınlı 2013); İzmir, Kütahya, Manisa, Uşak (Aydınlı and Ertorun 2015); listed from Turkey: Tanatmış (1999); Kazancı and Türkmen (2012).

##### Subgenus Labiobaetis Novikova & Kluge, 1987

**Comment.** *Labiobaetis* is either considered of generic rank (Barber-James et al. 2013) or a subgenus of *Baetis* (Novikova and Kluge 1987, Bauernfeind and Soldán 2011); see also the interesting discussion in Kluge (2015) [accessed October 15^th^ 2015].

**Baetis (Labiobaetis) atrebatinus Eaton, 1870**

**Distribution in Turkey.** Manisa, Uşak (Aydınlı and Ertorun 2015).

**Baetis (Labiobaetis) balcanicus Müller-Liebenau & Soldán, 1981**

**Distribution in Turkey.** Balıkesir (Kazancı 1998a); listed from Turkey: Kazancı (2001b); Kazancı and Türkmen (2012).

**Baetis (Labiobaetis) tricolor Tshernova, 1928**

**Distribution in Turkey.** Diyarbakır (Koch 1985); Hatay (Koch 1988); Sivas (Kazancı 1998a); Balıkesir (Tanatmış 2000); Artvin, Erzurum, Sivas (Kazancı 2001a); Bursa (Tanatmış 2002); Erzincan, Tunceli (Kazancı 2009); listed from Turkey: Tanatmış (1999); Kazancı (2001b); Kazancı and Türkmen (2012).

**Comment.** Occurrence of potamalic *Baetis tricolor* in high mountain streams (Erzincan, Tunceli at between 1000–1500 m a.s.l.) is rather doubtful. In the larval stage, usually not separable from *Baetis calcaratus* Keffermüller, 1972. A re-examination of voucher specimens would be desirable.

##### Subgenus Rhodobaetis Jacob, 2003

**Baetis (Rhodobaetis) bisri Thomas & Dia, 1983**

**Distribution in Turkey.** Hakkari, Dicle River Basin, 1500 m, 10. 7. 1986, 3 larvae (Kazancı 2009).

**Comment.** Novikova (1987: 79) suggested the possible synonymy of *Baetis bisri* with *Baetis stipposus* Kluge, 1982 [presently considered a junior subjective synonym of *Baetis braaschi* Zimmermann, 1980]. However, Godunko et al. (2004: 165) considered *Baetis bisri* a well-characterized taxon easily separated from *Baetis braaschi* in the nymph stage by several morphological characters. No information was provided by Kazancı (2009) on characters for identification and several closely related taxa have subsequently been described from the neighbouring (adjacent) Taurus region. Occurrence of *Baetis bisri* in Turkey is not very likely and the record may in fact be based on any taxon of the subgenus Baetis (Rhodobaetis). The record from Hakkari has obviously been listed subsequently by Kazancı and Türkmen (2012) as *Baetis braaschi* (see below).

**Baetis (Rhodobaetis) braaschi Zimmermann, 1980**

**Distribution in Turkey.** Probably Hakkari (as *Baetis bisri*; Kazancı 2009). Listed from Turkey: Kazancı and Türkmen (2012).

**Comment.** For diagnostic characters and their variability see Sroka et al. (2012).

**Baetis (Rhodobaetis) gemellus Eaton, 1885**

**Distribution in Turkey.** Ankara, Bingöl, Erzurum, Yozgat (Kazancı 1984); Muğla (Kazancı et al. 1992); Bolu (Kazancı and Türkmen 2008a); Bolu (Kazancı and Türkmen 2008b); Giresun, Gümüşhane, Rize, Trabzon (Türkmen and Kazancı 2015); listed from Turkey: Tanatmış (1999); Kazancı (2001b); Kazancı and Türkmen (2012); Eastern Black Sea Basin (Türkmen and Kazancı 2013).

**Comment.** *Baetis gemellus* Eaton is considered an insufficiently known taxon, at present represented by the male lectotype (Kimmins 1960) only. Larval characters and distribution not known, probably restricted to Switzerland. According to Godunko et al. (2015) all previous records as *Baetis gemellus* from Turkey refer in fact to *Baetis vadimi* Godunko, Palatov and Martynov, 2015.

**Baetis (Rhodobaetis) macrospinosus Koch, 1985**

**Type country and locality.** Turkey, Dicle river, 100 m upstream Dicle Bridge (at Gözlü Köprü), province of Diyarbakır (Koch 1985).

**Distribution in Turkey.** Diyarbakır (Koch 1985); listed from Turkey: Tanatmış (1999); Kazancı (2001b); Kazancı and Türkmen (2012).

**Note.** Endemic to Turkey.

**Baetis (Rhodobaetis) milani Godunko, Prokopov & Soldán, 2004**

**Distribution in Turkey.** Balıkesir (Türkmen and Özkan 2011); Gümüşhane, Rize, Trabzon (Türkmen and Kazancı 2015); listed from Turkey: Kazancı and Türkmen (2012); Eastern Black Sea Basin (Türkmen and Kazancı 2013).

**Comment.** According to Godunko et al. (2015: 196) the record from Balıkesir is doubtful and distribution in Anatolia needs confirmation.

**Baetis (Rhodobaetis) pseudogemellus Soldán, 1977**

**Distribution in Turkey.** Siirt (Kazancı 2009); listed from Turkey: Kazancı and Türkmen (2012).

**Comment.** Occurrence of *Baetis pseudogemellus* in Turkey is extremely unlikely (Godunko et al. 2015: 196), but several rather similar taxa of the subgenus Rhodobaetis occur in this region. A re-examination of voucher specimens from Siirt would be desirable.

**Baetis (Rhodobaetis) rhodani (Pictet, 1843)**

**Distribution in Turkey.** Antalya, Osmaniye (Puthz 1972); Elazığ (Berker 1981); Ankara, Balıkesir, Bayburt, Bingöl, Erzurum, Hakkari, Kars, Muş, Van (Kazancı 1984); Hatay (Koch 1988); Ankara, Bilecik, Bolu, Bursa, Eskişehir, Kütahya, Sakarya (Tanatmış 1995); Çanakkale, Edirne, İstanbul, Kırklareli, Tekirdağ (Tanatmış 1997); Balıkesir, Kütahya (Tanatmış 2000); Konya (Kazancı 2001b); Bursa, Kütahya (Tanatmış 2002); Bolu, Karabük, Kastamonu, Zonguldak (Tanatmış 2004a); Kastamonu, Sinop (Tanatmış 2004b); Balıkesir, Çanakkale (Narin and Tanatmış 2004); Sinop (Ertorun and Tanatmış 2004); Bartın (Tanatmış and Ertorun 2006); Düzce, Zonguldak (Tanatmış 2007); Ankara (Kazancı and Girgin 2008); Bolu (Kazancı and Türkmen 2008a); Bolu (Kazancı and Türkmen 2008b); Sinop (Tanatmış and Ertorun, 2008); Çankırı (Kazancı 2009); Balıkesir (Türkmen and Özkan 2011); Afyon, Konya (Özyurt and Tanatmış 2011); Tokat (Kazancı et al. 2012); Malatya (Aydınlı 2013); Giresun, Gümüşhane, Rize, Trabzon (Türkmen and Kazancı 2015); İzmir, Kütahya, Manisa, Uşak (Aydınlı and Ertorun 2015); listed from Turkey: Tanatmış (1999); Kazancı and Türkmen (2012); Eastern Black Sea Basin (Türkmen and Kazancı 2013).

**Comment.** Taxonomy of the *Baetis rhodani* species-group *sensu* Müller-Liebenau (1969) is rather complicated and numerous new species have been described in the more recent past. For a redescription and designation of neotype see Gattolliat and Sartori (2008).

**Baetis (Rhodobaetis) vadimi Godunko, Palatov & Martynov, 2015**

**Type country and locality.** Turkey, Unnamed brook, small right-side tributary of upper part of Fırtına Deresi (Kaçkar Mountains), district of Ardeşen, Rize province (Godunko et al. 2015).

**Distribution in Turkey.** Rize (Godunko et al. 2015).

**Comment.** Various earlier records as *Baetis gemellus* may in fact represent Baetis (Rhodobaetis) vadimi (see *Comment* above).

##### Subgenus Nigrobaetis Novikova & Kluge, 1987

**Comment.** *Nigrobaetis* is either considered of generic rank (Barber-James et al. 2013) or a subgenus of *Baetis* (Novikova and Kluge 1987, Bauernfeind and Soldán 2011). It is not clear, whether *Alainites* Waltz and McCafferty, 1994, should be treated as a distinct genus-group taxon (e.g. Barber-James et al. 2013) or included in *Labiobaetis* (e.g. Kluge 2015; accessed October 15^th^ 2015).

**Baetis (Nigrobaetis) digitatus Bengtsson, 1912**

**Distribution in Turkey.** Bolu, Muş, Sivas (Kazancı 1984); Bolu, Zonguldak (Tanatmış 2004a); Kastamonu, Sinop (Tanatmış 2004b); Balıkesir (Narin and Tanatmış 2004); Sinop (Ertorun and Tanatmış 2004); Bartın (Tanatmış and Ertorun 2006); Düzce, Zonguldak (Tanatmış 2007); listed from Turkey (Tanatmış 1999; Kazancı 2001b; Kazancı and Türkmen 2012).

**Comment.** Taxonomy of *Baetis digitatus* is insufficiently known and specific identity of southern (Mediterranean) and southeastern (Turkey, Caucasus region) representatives probably questionable. A series of reared material from Turkey would be especially interesting towards solving this question.

**Baetis (Nigrobaetis) gracilis Bogoescu & Tabacaru, 1957**

**Distribution in Turkey.** Balıkesir (Tanatmış 2000); Balıkesir (Kazancı 2001b); Balıkesir, Bursa (Tanatmış 2002); Karabük, Zonguldak (Tanatmış 2004a); listed from Turkey: Kazancı and Türkmen (2012).

**Baetis (Nigrobaetis) kars Thomas & Kazancı, 1989**

**Type country and locality.** Turkey, Kızılsu (the type locality is a stream and is located in the province of Şırnak) (Kazancı and Thomas 1989).

**Distribution in Turkey.** Hakkari, Kars, Şırnak (Kazancı and Thomas 1989); listed from Turkey: Tanatmış (1999); Kazancı (2001b); Kazancı and Türkmen (2012).

**Baetis (Nigrobaetis) muticus (Linnaeus, 1758)**

**Distribution in Turkey.** Bingöl, Van (Kazancı 1984); Muğla (Kazancı et al. 1992); Ankara, Bilecik, Bolu, Bursa, Eskişehir, Kütahya, Sakarya (Tanatmış 1995); İstanbul, Kırklareli, Tekirdağ (Tanatmış 1997); Balıkesir, Kütahya (Tanatmış 2000); Artvin, Erzincan, Erzurum, Kars (Kazancı 2001a); Kütahya (Tanatmış 2002); Bolu, Karabük, Kastamonu, Zonguldak (Tanatmış 2004a); Kastamonu, Sinop (Tanatmış 2004b); Sinop (Ertorun and Tanatmış 2004); Bartın (Tanatmış and Ertorun 2006); Düzce, Zonguldak (Tanatmış 2007); Bolu (Kazancı and Türkmen 2008a); Bolu (Kazancı and Türkmen 2008b); Sinop (Tanatmış and Ertorun 2008); Balıkesir (Türkmen and Özkan 2011); Afyon, Konya (Özyurt and Tanatmış 2011); Giresun, Rize, Trabzon (as *A. muticus*) (Türkmen and Kazancı 2015); Kütahya, Manisa (Aydınlı and Ertorun 2015); listed from Turkey: Tanatmış (1999); Kazancı (2001b); Kazancı and Türkmen (2012); Eastern Black Sea Basin (Türkmen and Kazancı 2013).

**Baetis (Nigrobaetis) niger (Linnaeus, 1761)**

**Distribution in Turkey.** Samsun (Kazancı 1984); Sivas (Koch 1985); Ankara (Kazancı 2001b); Manisa (Aydınlı and Ertorun 2015); listed from Turkey: Tanatmış (1999); Kazancı and Türkmen (2012).

#### Genus *Centroptilum* Eaton, 1869

***Centroptilum luteolum* (O.F. Müller, 1776)**

**Distribution in Turkey.** Ankara (Kazancı 1984); Sivas (Koch 1985); Ankara, Eskişehir (Tanatmış 1995); Çanakkale (Tanatmış 1997); Kütahya (Tanatmış 2000); Kütahya (Tanatmış 2002); Bolu, Karabük, Kastamonu, Zonguldak (Tanatmış 2004a); Kastamonu, Sinop (Tanatmış 2004b); Balıkesir (Narin and Tanatmış 2004); Sinop (Ertorun and Tanatmış 2004); Bartın (Tanatmış and Ertorun 2006); Zonguldak (Tanatmış 2007); Balıkesir (Türkmen and Özkan 2011); Afyon, Konya (Özyurt and Tanatmış 2011); Kütahya, Manisa (Aydınlı and Ertorun 2015); listed from Turkey: Tanatmış (1999); Kazancı (2001b); Kazancı and Türkmen (2012).

#### Genus *Cloeon* Leach, 1815

***Cloeon dipterum* (Linnaeus, 1761)**

**Distribution in Turkey.** Zonguldak (Verrier 1955); Afyon, Ankara, Ardahan, Erzurum, Nevşehir (Kazancı 1984); Ankara (Koch 1985); Hatay, Şanlıurfa (Koch 1988); Eskişehir, Kütahya, Sakarya (Tanatmış 1995); Edirne, İstanbul, Kırklareli, Tekirdağ (Tanatmış 1997); Balıkesir (Tanatmış 2000); Balıkesir, Bursa, Kütahya (Tanatmış 2002); Bolu, Karabük, Zonguldak (Tanatmış 2004a); Kastamonu (Tanatmış 2004b); Balıkesir, Çanakkale (Narin and Tanatmış 2004); Sinop (Ertorun and Tanatmış, 2004); Bartın (Tanatmış and Ertorun 2006); Düzce, Zonguldak (Tanatmış 2007); Bolu, Sakarya (Taşdemir et al. 2008); Konya (Topkara et al. 2009); Balıkesir (Türkmen and Özkan 2011); Afyon, Konya (Özyurt and Tanatmış 2011); Malatya (Aydınlı 2013); İzmir, Manisa, Uşak (Aydınlı and Ertorun 2015); listed from Turkey: Tanatmış (1999); Kazancı (2001b); Kazancı and Türkmen (2012).

***Cloeon simile* Eaton, 1870**

**Distribution in Turkey.** Ankara, Erzincan, Kırşehir (Kazancı 1984); Bursa (Tanatmış 1995); Balıkesir (Tanatmış 2000); Bolu, Kırşehir (Kazancı 2001b); Kütahya (Tanatmış 2002); Balıkesir (Narin and Tanatmış 2004); Malatya (Aydınlı 2013); Manisa (Aydınlı and Ertorun 2015); listed from Turkey: Tanatmış (1999); Kazancı and Türkmen (2012).

#### Genus *Procloeon* Bengtsson, 1915

***Procloeon bifidum* (Bengtsson, 1912)**

**Distribution in Turkey.** Eskişehir (Kazancı 1984); Kırklareli, Tekirdağ (Tanatmış 1997); Balıkesir (Tanatmış 2000); Kütahya (Tanatmış 2002); Bolu, Zonguldak (Tanatmış 2004a); Sinop (Tanatmış 2004b); Balıkesir (Narin and Tanatmış 2004); Sinop (Ertorun and Tanatmış 2004); Bartın (Tanatmış and Ertorun 2006); Düzce, Zonguldak (Tanatmış 2007); listed from Turkey: Tanatmış (1999); Kazancı (2001b); Kazancı and Türkmen (2012).

***Procloeon nana* (Bogoescu, 1951)**

**Distribution in Turkey.** Ağrı (as *Centroptilum nanum*; Kazancı 1984); listed from Turkey: Tanatmış (1999); Kazancı (2001b); Kazancı and Türkmen (2012).

**Comment.** Frequently placed in the subgenus (originally genus) *Pseudocentroptilum* Bogoescu, 1947. Very similar to (or conspecific with) *Pseudocentroptilum macronyx* (Kluge and Novikova 1992), see discussion in Bauernfeind and Soldán (2012: 212) and Kluge (2015).

***Procloeon pennulatum* (Eaton, 1870)**

**Distribution in Turkey.** Ağrı, Ankara, Erzurum (as *Centroptilum pennulatum*; Kazancı 1984); Şanlıurfa (as *Centroptilum pennulatum*; Koch 1988); Çanakkale, Kırklareli (Tanatmış 1997); Balıkesir, Bursa, Kütahya (Tanatmış 2000); Balıkesir, Kütahya (Tanatmış 2000); Erzurum (Kazancı 2001a); Ankara, Çankırı (Kazancı 2001b); Balıkesir, Bursa, Kütahya (Tanatmış 2002); Bolu, Karabük, Zonguldak (Tanatmış 2004a); Kastamonu (Tanatmış 2004b); Bartın (Tanatmış and Ertorun 2006); Düzce, Zonguldak (Tanatmış 2007); Afyon, Konya (Özyurt and Tanatmış 2011); Manisa (Aydınlı and Ertorun 2015); listed from Turkey: Tanatmış (1999); Kazancı and Türkmen (2012).

**Comment.** Frequently placed in the subgenus (originally genus) *Pseudocentroptilum* Bogoescu, 1947.

***Procloeon pulchrum* (Eaton, 1885)**

**Distribution in Turkey.** Bingöl, Kars (as *Centroptilum pulchrum*; Kazancı 1984); Diyarbakır (as Centroptilum ? pulchrum: Koch 1985); Şanlıurfa (as Centroptilum ? pulchrum: Koch 1988); Zonguldak (Tanatmış 2004a); Kastamonu, Sinop (Tanatmış 2004b); Sinop (Ertorun and Tanatmış 2004); Düzce (Tanatmış 2007); Sinop (Tanatmış and Ertorun 2008); listed from Turkey: Tanatmış (1999); Kazancı (2001b); Kazancı and Türkmen (2012).

**Comment.** Frequently placed in the subgenus (originally genus) *Pseudocentroptilum* Bogoescu, 1947.

#### Genus *Pseudocentroptiloides* Jacob, 1986

***Pseudocentroptiloides shadini* (Kazlauskas, 1964)**

**Distribution in Turkey.** Çankırı, Kırşehir (Kazancı 2001b); listed from Turkey: Kazancı and Türkmen (2012).

**Comment.** Has been placed in *Psammonella* Glazaczow (in Jacob and Glazaczow), 1986 by Kluge and Novikova (1992), but see discussion in Kluge (2015; accessed October 15^th^ 2015).

### Family ISONYCHIIDAE Burks, 1953

#### Genus *Isonychia* Albarda, 1878

***Isonychia ignota* Walker, 1853**

**Material.** Yalova, brook I Köseler, Koruköy, male imago, female imago, 30.5.1992, H. Malicky leg., 38°50'N, 27°10'E (NMW).

**Distribution in Turkey.** Samsun, Zonguldak (Kazancı 1986a); Eskişehir (Tanatmış 1995); İstanbul (Tanatmış 1997); Balıkesir (Tanatmış 2000); Erzincan (Kazancı 2001a); Ankara, Balıkesir, Muğla (Kazancı 2001b); Balıkesir (Tanatmış 2002); Karabük, Zonguldak (Tanatmış 2004a); Kastamonu (Tanatmış 2004b); Balıkesir, Çanakkale (Narin and Tanatmış 2004); Sinop (Ertorun and Tanatmış 2004); Bartın (Tanatmış and Ertorun 2006); Zonguldak (Tanatmış 2007); listed from Turkey: Tanatmış (1999); Kazancı and Türkmen (2012).

### Family OLIGONEURIIDAE Ulmer, 1914

#### Genus *Oligoneuriella* Ulmer, 1924

***Oligoneuriella magna* Bojková & Soldán in Sroka et al., 2015**

**Type country and locality.** Turkey, Kayseri, Zamantı Irmağı River, Eşelik (near Taşçı); 38°12'42.7"N / 35°50'31.1"E.

**Distribution in Turkey.** Kayseri (Sroka et al. 2015).

**Note.** Endemic to Turkey.

**Comment.** Imago not described.

***Oligoneuriella orontensis* Koch, 1980**

**Distribution in Turkey.** Hatay (Koch 1980); Diyarbakır (Koch 1985); Hatay (Koch 1988); Erzincan, Erzurum, Tunceli (Kazancı 2001a); listed from Turkey: Tanatmış (1999); Kazancı (2001b); Kazancı and Türkmen (2012).

**Comment.** Imago not described.

***Oligoneuriella pallida* (Hagen, 1855)**

**Distribution in Turkey.** Amasya (as *Oligoneuriella mikulskii* Sowa, 1961; Kazancı 1986a); listed from Turkey: Tanatmış (1999); Kazancı (2001b); Kazancı and Türkmen (2012).

***Oligoneuriella paulopilosa* Sroka in Sroka et al., 2015**

**Type country and locality.** Turkey, Adıyaman, Sürgü Çayı river near Gölbaşı; 37°50'10.2"N / 37°41'06.9"E (Sroka et al. 2015).

**Distribution in Turkey.** Adıyaman (Sroka et al. 2015).

**Note.** Endemic to Turkey.

**Comment.** Imago not described.

***Oligoneuriella pectinata* Bojková & Soldán in Sroka et al., 2015**

**Type country and locality.** Turkey, Adıyaman, right tributary of Göksu Çayı, Taşlıyazı (near Besni); 37°42'36.9"N / 37°56'16.0"E (Sroka et al. 2015).

**Distribution in Turkey.** Adıyaman (Sroka et al. 2015).

**Note.** Endemic to Turkey.

***Oligoneuriella rhenana* (Imhoff, 1852)**

**Distribution in Turkey.** Kırklareli (Kazancı 1986a); Bilecik, Bolu, Eskişehir (Tanatmış 1995); Kırklareli (Tanatmış 1997); Balıkesir, Kütahya (Tanatmış 2000); Balıkesir, Muğla, Kırklareli (Kazancı 2001b); Balıkesir, Bursa, Kütahya (Tanatmış 2002); Bolu, Karabük, Zonguldak (Tanatmış 2004a); Kastamonu, Sinop (Tanatmış 2004b); Balıkesir (Narin and Tanatmış 2004); Sinop (Ertorun and Tanatmış 2004); Bartın (Tanatmış and Ertorun 2006); Sinop (Tanatmış and Ertorun 2008); Bolu (Kazancı and Türkmen 2008a); Bolu (Kazancı and Türkmen 2008b); Tokat (Kazancı et al. 2012); listed from Turkey: Tanatmış (1999); Kazancı and Türkmen (2012).

***Oligoneuriella tskhomelidzei* Sowa & Zosidze, 1973**

**Distribution in Turkey.** Van (as *Oligoneuriella baskale* Soldán and Landa 1977; Soldan and Landa 1977); Artvin, Erzincan (as *Oligoneuriella zanga* Soldán and Landa; Kazancı 2001a); Kars, Van (as *Oligoneuriella baskale* Soldán and Landa; Kazancı 2009); listed from Turkey: Kazancı (2001b); Kazancı and Türkmen (2012).

**Comment.** Imago of *Oligoneuriella tskhomelidzei* not described. Both, *Oligoneuriella baskale* Soldán and Landa, 1977 and *Oligoneuriella zanga* Soldán and Landa, 1977 have been considered to represent junior subjective synonyms of *Oligoneuriella tskhomelidzei* Sowa and Zosidze, 1973 by Kluge (2004).

### Family HEPTAGENIIDAE Needham in Needham & Betten, 1901

#### Genus *Ecdyonurus* Eaton, 1865

**Comment.** For use of the name *Ecdyonurus* see Bauernfeind and Haybach (2012) and ICZN (2015).

***Ecdyonurus bimaculatus* Tanatmış & Haybach, 2010**

**Type country and locality.** Turkey, Emet Stream (Emet Stream is located in the Harmancık – Dursunbey Road 24.km, Hopanlar village, the province of Balıkesir) (Tanatmış and Haybach 2010).

**Distribution in Turkey.** Balıkesir, Bursa, Karabük, Kütahya, Sinop, Zonguldak (Tanatmış and Haybach 2010).

**Note.** Endemic to Turkey.

**Comment.** Tentatively placed in *Ecdyonurus* by Tanatmış and Haybach (2010), very similar to *Afghanurus vicinus* Demoulin, 1964 (larva not described) and *Afronurus*? sp. 1 of Demoulin (1963: p. 37, imago not described). For a discussion of taxonomic concepts for *Ecdyonurus* Eaton see Bauernfeind and Soldán (2012: 251) and Bauernfeind and Haybach (2012).

##### Subgenus Ecdyonurus Eaton, 1865

**Ecdyonurus (Ecdyonurus) aurantiacus (Burmeister, 1839)**

**Distribution in Turkey.** Erzurum (Kazancı 2009); listed from Turkey: Kazancı and Türkmen (2012).

**Ecdyonurus (Ecdyonurus) autumnalis Braasch, 1980**

**Distribution in Turkey.** Artvin (Kazancı 2001b); Artvin (Kazancı 2001a); listed from Turkey: Tanatmış (1999); Kazancı and Türkmen (2012).

**Comment.** Larva not described.

**Ecdyonurus (Ecdyonurus) dispar (Curtis, 1834)**

**Distribution in Turkey.** Ankara (as *Ecdyonurus fluminum* Pictet, 1843; Geldiay 1949); Erzurum, Hakkari, Kars (Kazancı 2001b); Kütahya (Tanatmış 2002); Karabük, Zonguldak (Tanatmış 2004a); Kastamonu (Tanatmış 2004b); Balıkesir (Narin and Tanatmış 2004); Sinop (Ertorun and Tanatmış 2004); Bartın (Tanatmış and Ertorun 2006); Hakkari (Kazancı 2009); Manisa (Aydınlı and Ertorun 2015); listed from Turkey: Tanatmış (1999); Kazancı and Türkmen (2012).

**Ecdyonurus (Ecdyonurus) insignis (Eaton, 1870)**

**Distribution in Turkey.** İstanbul, Kırklareli, Tekirdağ (Tanatmış 1997); Balıkesir, Kütahya (Tanatmış, 2000); Sivas (Kazancı 2001b); Kütahya (Tanatmış 2002); Bolu, Karabük, Zonguldak (Tanatmış 2004a); Kastamonu, Sinop (Tanatmış 2004b); Bartın (Tanatmış and Ertorun 2006); Düzce, Zonguldak (Tanatmış 2007); Bolu (Kazancı and Türkmen 2008a); Bolu (Kazancı and Türkmen 2008b); listed from Turkey: Tanatmış (1999); Kazancı and Türkmen (2012).

**Ecdyonurus (Ecdyonurus) macani Thomas & Sowa, 1970**

**Distribution in Turkey.** Giresun (Türkmen and Kazancı 2015); listed from Turkey: Eastern Black Sea Basin (Türkmen and Kazancı 2013).

**Comment.** Occurrence of *Ecdyonurus macani* Thomas and Sowa in Turkey is probably doubtful and a re-examination of material would be advisable.

**Ecdyonurus (Ecdyonurus) ornatipennis Tshernova, 1938**

**Distribution in Turkey.** Muş (Braasch 1981); Amasya (Kazancı and Braasch 1988); listed from Turkey: Tanatmış (1999); Kazancı (2001b); Kazancı and Türkmen (2012).

**Ecdyonurus (Ecdyonurus) russevi Braasch & Soldán, 1985**

**Distribution in Turkey.** Balıkesir (Kazancı 2001b); listed from Turkey: Kazancı and Türkmen (2012).

**Comment.** First larval description (and redescription of imaginal stages) was given by Godunko et al. (2015).

**Ecdyonurus (Ecdyonurus) submontanus Landa, 1969**

**Distribution in Turkey.** Ardahan (Kazancı 2009); listed from Turkey: Kazancı and Türkmen (2012).

**Comment.** Occurrence of *Ecdyonurus submontanus* Landa in Turkey rather questionable, a re-examination of voucher specimens would be advisable.

**Ecdyonurus (Ecdyonurus) starmachi Sowa, 1971**

**Distribution in Turkey.** Bolu (Kazancı and Türkmen 2008a); Bolu (Kazancı and Türkmen 2008b); Giresun, Rize, (Türkmen and Kazancı 2015); listed from Turkey: Kazancı and Türkmen (2012); Eastern Black Sea Basin (Türkmen and Kazancı 2013).

**Ecdyonurus (Ecdyonurus) venosus (Fabricius, 1775)**

**Distribution in Turkey.** Bolu, Bursa, Eskişehir, Kütahya (Tanatmış 1995); Çanakkale, Kırklareli, Tekirdağ (Tanatmış 1997); Balıkesir (Tanatmış 2000); Ankara, Çankırı, Eskişehir, Konya (Kazancı 2001b); Ankara (Kazancı and Girgin 2008); Bolu (Kazancı and Türkmen 2008a); Bolu (Kazancı and Türkmen 2008b); Erzincan (Kazancı 2009); listed from Turkey: Tanatmış (1999); Kazancı and Türkmen (2012).

**Comment.** Occurrence in Turkey rather doubtful, probably based on misidentification. A re-examination of voucher specimens would be advisable.

##### Subgenus Helvetoraeticus Bauernfeind & Soldán, 2012

**Ecdyonurus (Helvetoraeticus) helveticus Eaton, 1883**

**Distribution in Turkey.** Ankara, Bolu, Eskişehir (Kazancı 2001b); Bolu (Tanatmış 2004a); Düzce (Tanatmış 2007); Giresun, Rize (Türkmen and Kazancı 2015); Kütahya (Aydınlı and Ertorun 2015); listed from Turkey: Kazancı and Türkmen (2012); Eastern Black Sea Basin (Türkmen and Kazancı 2013).

**Comment.** Occurrence of the alpine taxon *Ecdyonurus helveticus* Eaton in Turkey is rather doubtful and most probably based on misidentification. A re-examination of material would be advisable.

**Ecdyonurus (Helvetoraeticus) picteti (Meyer-Dür, 1864)**

**Distribution in Turkey.** Giresun, Rize (Türkmen and Kazancı 2015); listed from Turkey: Eastern Black Sea Basin (Türkmen and Kazancı 2013).

**Comment.** Occurrence of the alpine taxon *Ecdyonurus picteti* (Meyer-Dür) in Turkey is rather doubtful and most probably based on misidentification. A re-examination of material would be advisable.

#### Genus *Electrogena* Zurwerra & Tomka, 1985

***Electrogena affinis* (Eaton, 1883)**

**Distribution in Turkey.** Balıkesir (Türkmen and Özkan 2011); Giresun, Rize, Trabzon (Türkmen and Kazancı 2015); listed for Turkey: Eastern Black Sea Basin (Türkmen and Kazancı 2013).

***Electrogena anatolica* (Kazanci & Braasch, 1986)**

**Type country and locality.** Turkey, Ardahan (Kazancı and Braasch 1986).

**Distribution in Turkey.** Ankara, Ardahan, Bingöl, Bolu, Kars (as *Ecdyonurus anatolicus*; Kazancı and Braasch 1986); Ankara, Ardahan, Erzurum, Hakkari (Kazancı 1990b); Ardahan, Kars (Kazancı 2001a); Kars (Kazancı 2009); listed from Turkey: Tanatmış (1999); Kazancı (2001b); Kazancı and Türkmen (2012).

**Note.** Endemic to Turkey.

**Comment.** Larva not described.

***Electrogena antalyensis* (Braasch & Kazanci in Kazancı & Braasch, 1986)**

**Type country and locality.** Turkey, Burçak Village (the type locality is located in the province of Ankara) (Kazancı and Braasch 1986).

**Material.** Samsun, Sahinkaya, male imago, 6.6.1992, Malicky leg., 40°11'N, 25°46'E (NMW).

**Distribution in Turkey.** Ankara, Antalya, Çorum, Yozgat (as *Ecdyonurus antalyensis*; Kazancı and Braasch 1986); Ankara, Bolu (Kazancı 1990b); Ankara, Antalya, Eskişehir, Kütahya (Belfiore et al. 2000); Kırklareli (Kazancı 2001b); Kütahya (Tanatmış 2002); Bolu (Tanatmış 2004a); Manisa (Aydınlı and Ertorun 2015); listed from Turkey: Tanatmış (1999); Kazancı and Türkmen (2012).

**Comment.** For description of larva (and redescription of imaginal stages) see Belfiore et al. (2000).

***Electrogena boluensis* Kazanci, 1990b**

**Type country and locality.** Turkey, Bolu-Gerede road, 10 km to Gerede (the type locality is located in the province of Bolu) (Kazancı 1990b).

**Distribution in Turkey.** Bolu (Kazancı 1990b); listed from Turkey: Tanatmış (1999); Kazancı (2001b); Kazancı and Türkmen (2012).

**Note.** Endemic to Turkey.

**Comment.** Larva not described.

***Electrogena dirmil* Kazanci, 1990b**

**Type country and locality.** Turkey, Dirmil Pass (the type locality is located in the district of Fethiye, the province of Muğla) (Kazancı 1990b).

**Distribution in Turkey.** Muğla (Kazancı 1990b); Muğla (Kazancı et al. 1992); listed from Turkey: Tanatmış (1999); Kazancı (2001b); Kazancı and Türkmen (2012).

**Note.** Endemic to Turkey.

**Comment.** Larva not described.

***Electrogena hakkarica* (Kazanci, 1986b)**

**Type country and locality.** Turkey, Güzereş Köyü (Güzereş Köyü is a village and is located in the district of Çukurca, the province of Hakkari) (Kazancı 1986b).

**Distribution in Turkey.** Hakkari (as *Ecdyonurus hakkaricus*; Kazancı 1986b); Rize (Kazancı 2001b); listed from Turkey: Tanatmış (1999); Kazancı and Türkmen (2012).

**Note.** Endemic to Turkey.

**Comment.** Larva not described.

***Electrogena lateralis* (Curtis, 1834)**

**Distribution in Turkey.** Ankara (as *Ecdyonurus lateralis*; Kazancı 1985a); Ankara, Bilecik, Bolu, Eskişehir, Kütahya (Tanatmış 1995); İstanbul, Kırklareli, Tekirdağ (Tanatmış 1997); Balıkesir, Bursa (Tanatmış 2000); Çorum, Yozgat (Kazancı 2001b); listed from Turkey: Tanatmış (1999); Kazancı and Türkmen (2012).

***Electrogena monticola* (Braasch, 1980)**

**Distribution in Turkey.** Tunceli (Kazancı 1990b); listed from Turkey: Tanatmış (1999); Kazancı (2001b); Kazancı and Türkmen (2012).

**Comment.** Originally as *Ecdyonurus monticolus*. Larva not described.

***Electrogena necatii* (Kazanci, 1987a)**

**Type country and locality.** Turkey, Akyarma Pass (Akyarma Geçidi is a pass and is located between Bolu-Ankara Road; Kazancı 1987a).

**Distribution in Turkey.** Ankara, Bolu (as *Ecdyonurus necatii*; Kazancı 1987a); Ankara (Kazancı and Girgin 2008); listed from Turkey: Tanatmış (1999); Kazancı (2001b); Kazancı and Türkmen (2012).

**Note.** Endemic to Turkey

**Comment.** Larva not described.

***Electrogena pseudaffinis* (Braasch, 1980)**

**Distribution in Turkey.** Trabzon (as *Ecdyonurus pseudaffinis*; Kazancı and Braasch 1988); listed from Turkey: Tanatmış (1999); Kazancı (2001b); Kazancı and Türkmen (2012).

***Electrogena quadrilineata* (Landa, 1969)**

**Distribution in Turkey.** Giresun, Rize, Trabzon (Türkmen and Kazancı 2015); listed from Turkey: eastern Black Sea Basin (Türkmen and Kazancı 2013).

**Comment.** *Electrogena quadrilineata* has so far only been recorded from a few localities in Central Europe and occurrence in Turkey is rather questionable. For confusing taxa see Bauernfeind and Soldán (2012), a redescription from type material has been provided by Kłonowska-Olejnik (2004).

***Electrogena ressli* (Braasch, 1981)**

**Type country and locality.** Turkey, Van Gölü (the type locality is located in the province of Muş) (Braasch 1981).

**Distribution in Turkey.** Muş (as *Ecdyonurus ressli*; Braasch 1981); Erzincan, Erzurum, Muş, Tunceli (Kazancı 2009); listed from Turkey: Tanatmış (1999); Kazancı (2001b); Kazancı and Türkmen (2012).

**Comment.** Larva not described.

#### Genus *Afronurus* Lestage, 1924

***Afronurus kugleri* Demoulin, 1973**

**Material.** Yalova, brook I Köseler, Koruköy, male subimago, 30.5.1992, H. Malicky leg., 38°50'N, / 27°14'E (NMW).

**Distribution in Turkey.** Hatay (Koch 1988); Ankara, Bingöl, Bolu, Elazığ, Muş (Kazancı and Braasch 1988); listed from Turkey: Tanatmış (1999); Kazancı (2001b); Kazancı and Türkmen (2012).

**Comment.** Taxonomic position of palaearctic representatives of *Afronurus* (*Afronurus kugleri*, *Afronurus madli* and *Afronurus zebratus*) remains uncertain and rather provisional (included in *Electrogena* by Kluge 2004, Phyl. Syst. Eph., 184), not considered in Webb and McCafferty 2008 (Can. J. Arthropod Identif. 7: 2-3). For generic placement in a new genus see Yanai et al. (2016).

***Afronurus madli* Kazanci, 1992**

**Type country and locality.** Turkey, Karacadağ (the type locality is located in the province of Diyarbakır) (Kazancı 1992).

**Distribution in Turkey.** Diyarbakır (Kazancı 1992); listed from Turkey: Tanatmış (1999); Kazancı (2001b); Kazancı and Türkmen (2012).

**Comment.** Larva not described. Taxonomic position of palaearctic representatives of *Afronurus* (*Afronurus kugleri*, *Afronurus madli* and *Afronurus zebratus*) remains uncertain and rather provisional (included in *Electrogena* by Kluge 2004, Phyl. Syst. Eph., 184), not considered in Webb and McCafferty 2008 (Can. J. Arthropod Identif. 7: 2–3). For generic placement see Yanai et al. (2016).

**Note.** Endemic to Turkey.

#### Genus *Epeorus* Eaton, 1881

##### Subgenus Caucasiron Kluge, 1997

**Epeorus (Caucasiron) alpestris (Braasch, 1979)**

**Distribution in Turkey.** Artvin, Kars (as *Iron alpestris*; Kazancı 1986a); Artvin, Kars (as *Iron alpestris*; Kazancı 2001a); listed from Turkey: Tanatmış (1999); Kazancı (2001b); Kazancı and Türkmen (2012).

**Comment.** Taxonomy follows Kluge (1997a). Webb and McCafferty (2008, Can. J. Arthropod Ident. 7: 1-55) did not recognize subgenera or species-groups within *Epeorus*.

**Epeorus (Caucasiron) caucasicus (Tshernova, 1938)**

**Distribution in Turkey.** Van (as *Iron caucasicus*; Braasch 1981); Artvin, Erzincan, Erzurum (Kazancı 1986a); Adıyaman (as *Iron caucasicus*; Koch 1988); Artvin, Erzincan, Erzurum (Kazancı 2001a); Erzincan, Erzurum, Hakkari, Tunceli (Kazancı 2009); Giresun, Gümüşhane, Rize, Trabzon (Türkmen and Kazancı 2015); listed from Turkey: Tanatmış (1999); Kazancı (2001b); Kazancı and Türkmen 2012); Eastern Black Sea Basin (Türkmen and Kazancı 2013).

**Comment.** Taxonomy follows Kluge (1997a). *Cinygma caucasica* Tshernova, 1938 has been designated type species of the subgenus Caucasiron Kluge, 1997.

**Epeorus (Caucasiron) fuscus (Sinitshenkova, 1976)**

**Distribution in Turkey.** Erzincan (Kazancı 2009).

**Epeorus (Caucasiron) longimaculatus (Braasch, 1980)**

**Distribution in Turkey.** Bursa (as *Iron longimaculatus*; Kazancı and Braasch 1988); listed from Turkey: Tanatmış (1999); Kazancı (2001b); Kazancı and Türkmen (2012).

**Epeorus (Caucasiron) magnus (Braasch, 1978)**

**Distribution in Turkey.** Rize (as *Iron magnus*; Kazancı 2009); listed from Turkey: Kazancı and Türkmen (2012).

**Epeorus (Caucasiron) nigripilosus (Sinitshenkova, 1976)**

**Distribution in Turkey.** Hakkari (as *Iron nigripilosus*; Kazancı 2001b); Erzincan, Şırnak (Kazancı 2009); listed from Turkey: Kazancı and Türkmen (2012).

**Epeorus (Caucasiron) znojkoi (Tshernova, 1938 [sub *Iron znojkoi*])**

**Distribution in Turkey.** Van (as *Iron znojkoi*; Braasch, 1981); Giresun, Rize, Trabzon (Türkmen and Kazancı 2015); listed from Turkey: Eastern Black Sea Basin (Türkmen and Kazancı 2013).

**Comment.** Not to be confused with *Ecdyonurus znojkoi* Tshernova, 1938 (presently placed in *Rhithrogena*). For the generic placement see Kluge (1997a: 233). *Epeorus znojkoi* *sensu* Braasch (1978; larva) nec Tshernova (1938) represents in fact *Epeorus zaitzevi* Tshernova, 1981 (see Sartori 1992).

##### Subgenus Epeorus Eaton, 1881

**Epeorus (Epeorus) assimilis Eaton, 1885**

**Distribution in Turkey.** Kırklareli (as *Epeorus sylvicola*; Tanatmış 1997); Ankara (sub *Epeorus sylvicola*; Kazancı 2001b); Ankara (as *Epeorus sylvicola*; Kazancı and Girgin 2008); Giresun, Gümüşhane, Rize, Trabzon (as *Epeorus sylvicola*; Türkmen and Kazancı 2015); listed from Turkey: Tanatmış (1999); Kazancı and Türkmen (2012); Eastern Black Sea Basin (Türkmen and Kazancı 2013).

**Comment.** Frequently considered a junior subjective synonym of Epeorus (Epeorus) sylvicola (Pictet, 1865), but see Thomas et al. (1999: 85).

**Epeorus (Epeorus) zaitzevi Tshernova, 1981**

**Distribution in Turkey.** Şanlıurfa (as *Epeorus zaitcevi* [injustified emendation (see Sartori 1992)]; Koch 1988); Ardahan, Bayburt, Erzurum, Hakkari (as *Epeorus zaitcevi* [injustified emendation (see Sartori 1992)]; Kazancı and Braasch 1988); Erzurum, Hakkari, Kars, Şırnak, Tunceli (Kazancı 2009); Giresun, Gümüşhane (Türkmen and Kazancı 2015); listed from Turkey: Tanatmış (1999); Kazancı (2001b); Kazancı and Türkmen (2012); Eastern Black Sea Basin (Türkmen and Kazancı 2013).

**Comment.** Larval characters of *Epeorus znojkoi* *sensu* Braasch (1978) refer in fact to Epeorus (Epeorus) zaitzevi Tshernova, 1981 (see Sartori 1992).

##### Subgenus Ironopsis Traver, 1935

**Epeorus (Ironopsis) alpicola (Eaton, 1871)**

**Distribution in Turkey.** Bursa, Eskişehir, Kütahya (Tanatmış 1995); Kütahya (Tanatmış 2000); Kütahya (Tanatmış 2002); Sinop (Ertorun and Tanatmış 2004); Giresun, Rize, Trabzon (Türkmen and Kazancı 2015); listed from Turkey: Tanatmış (1999); Kazancı (2001b); Kazancı and Türkmen (2012); Eastern Black Sea Basin (Türkmen and Kazancı 2013).

**Comment.** Taxonomy follows Kluge (1997a). Braasch (2006) proposed the new subgenus Alpiron Braasch for the European representatives of the subgenus Ironopsis Traver. Occurrence in Turkey somewhat doubtful, a re-examination of voucher specimens would be advisable.

#### Genus *Rhithrogena* Eaton, 1881

***Rhithrogena amseli* (Demoulin, 1964) [sub *Epeiron***]

**Distribution in Turkey.** Hakkari, Muş (Kazancı 2009); listed from Turkey: Kazancı and Türkmen (2012).

**Comment.** By most authors *Epeiron* Demoulin is currently considered to represent a junior synonym of *Rhithrogena* Eaton [cf. Kluge (1988: 304)]. Recently Kluge (2004: 195) reconsidered the generic status of *Epeiron*. Wang and McCafferty (2004) placed the taxon in the genus *Cinygmula* Mcdunnough, 1933. Occurrence in Turkey rather questionable, a re-examination of voucher specimens would be desirable.

***Rhithrogena anatolica* Kazancı, 1985b**

**Type country and locality.** Turkey, Kızılırmak River in Kırıkkale Province (Kazancı 1985b).

**Distribution in Turkey.** Erzurum, Kırıkkale, Siirt, Sivas (Kazancı 1985b); Erzurum (Kazancı 2001a); Ankara (Kazancı 2001b); listed from Turkey: Tanatmış (1999); Kazancı and Türkmen (2012).

**Note.** Endemic to Turkey.

**Comment.** Larva not described. Placed in *Epeiron* Demoulin 1964 by Kluge (2004).

***Rhithrogena beskidensis* Alba-Tercedor & Sowa, 1987**

**Distribution in Turkey.** Rize (Türkmen and Kazancı 2015); listed from Turkey: Eastern Black Sea Basin (Türkmen and Kazancı 2013).

**Comment.** So far considered to represent rather a west Palaearctic taxon, distribution on the Balkans and in Turkey probably questionable (Bauernfeind and Soldán 2012: 336).

***Rhithrogena braaschi* Jacob, 1974**

**Material.** Elazığ, Soğukpınar, E-Anatolia, male imago, female imago, 4.6.1992, H. Malicky leg., 38°25'N, / 39°15'E (NMW).

**Distribution in Turkey.** Bolu (Kazancı and Braasch 1988); Bolu (Tanatmış 2004a); listed from Turkey: Tanatmış (1999); Kazancı (2001b); Kazancı and Türkmen (2012).

***Rhithrogena caucasica* Braasch, 1979**

**Distribution in Turkey.** Hakkari (Kazancı and Braasch 1988); Erzincan, Erzurum (Kazancı 2001a); Bingöl (Kazancı 2009); listed from Turkey: Tanatmış (1999); Kazancı (2001b); Kazancı and Türkmen (2012).

***Rhithrogena expectata* Braasch, 1979**

**Distribution in Turkey.** Erzurum (Kazancı and Braasch 1988); Erzurum (Kazancı 2001a); listed from Turkey: Tanatmış (1999); Kazancı (2001b); Kazancı and Türkmen (2012).

***Rhithrogena germanica* Eaton, 1885**

**Distribution in Turkey.** Giresun, Rize, Trabzon (Türkmen and Kazancı 2015); listed from Turkey: Eastern Black Sea Basin (Türkmen and Kazancı, 2013).

**Comment.** Larvae are very difficult to separate from several representatives of the *Rhithrogena semicolorata* species-group and occurrence in Turkey probably doubtful (Bauernfeind and Soldán 2012: 344).

***Rhithrogena iranica* Braasch, 1983**

**Distribution in Turkey.** Muş (Kazancı and Braasch 1988); Erzurum (Kazancı 2001a); Van (Kazancı 2009); listed from Turkey: Tanatmış (1999); Kazancı (2001b); Kazancı and Türkmen, 2012).

**Comment.** Larva not described.

***Rhithrogena iridina kownackorum* Sowa & Zimmermann, 1975**

**Distribution in Turkey.** Gümüşhane (Kazancı and Braasch 1988); Erzincan, Kars (Kazancı 2009); Giresun, Rize, Trabzon (as *Rhithrogena iridina*; Türkmen and Kazancı 2015); listed from Turkey: Tanatmış (1999); Kazancı (2001b); Kazancı and Türkmen (2012); Eastern Black Sea Basin (Türkmen and Kazancı, 2013).

**Comment.** Larvae are very difficult to separate from several representatives of the *Rhithrogena semicolorata* species-group.

***Rhithrogena loyolaea* Navás, 1922**

**Distribution in Turkey.** Artvin (Kazancı 1985a); listed from Turkey: Tanatmış (1999); Kazancı (2001b); Kazancı and Türkmen (2012).

**Comment.** So far considered to represent a west-central Palaearctic taxon, distribution on the Balkans and in Turkey probably questionable (Bauernfeind and Soldán 2012: 368). For taxonomic characters see Kłonowska-Olejnik and Godunko (2003).

***Rhithrogena pontica* Sowa, Soldán & Kazanci, 1986**

**Type country and locality.** Turkey, stream 30 km south of Tortum (the type locality is located in the district of Tortum, the province of Erzurum) (Sowa et al. 1986).

**Distribution in Turkey.** Erzurum (Sowa et al. 1986); listed from Turkey: Tanatmış (1999); Kazancı (2001b); Kazancı and Türkmen (2012).

**Note.** Endemic to Turkey.

**Comment.** Larva not described.

***Rhithrogena potamalis* Braasch, 1979**

**Distribution in Turkey.** Kahramanmaraş (Kazancı 1986a); listed from Turkey: Tanatmış (1999); Kazancı (2001b); Kazancı and Türkmen (2012).

**Comment.** Imago not described.

***Rhithrogena puytoraci* Sowa & Degrange, 1987**

**Distribution in Turkey.** Giresun, Rize, Trabzon (Türkmen and Kazancı 2015); listed from Turkey: Eastern Black Sea Basin (Türkmen and Kazancı 2013).

**Comment.** So far considered to represent rather a central Palaearctic taxon, distribution in Turkey is probably questionable (Bauernfeind and Soldán 2012: 378).

***Rhithrogena semicolorata* (Curtis, 1834)**

**Distribution in Turkey.** Bayburt, Çankırı (Kazancı 1985a); Bilecik, Bursa, Eskişehir (Tanatmış 1995); Kırklareli, Tekirdağ (Tanatmış 1997); Giresun, Rize, Trabzon (Türkmen and Kazancı 2015); Kütahya (Aydınlı and Ertorun 2015); listed from Turkey: Tanatmış (1999); Kazancı (2001b); Kazancı and Türkmen (2012); Eastern Black Sea Basin (Türkmen and Kazancı 2013).

***Rhithrogena sublineata* Kazanci & Braasch, 1988**

**Type country and locality.** Turkey, Otluca (Otluca is a village and is located in the province of Hakkari) (Kazancı and Braasch 1988).

**Distribution in Turkey.** Hakkari (Kazancı and Braasch 1988); listed from Turkey: Tanatmış (1999); Kazancı (2001b); Kazancı and Türkmen (2012).

**Note.** Endemic to Turkey.

**Comment.** The taxon was described from a male subimago, larvae and imagines not known so far and generic placement doubtful.

***Rhithrogena tibialis* (Ulmer, 1920)**

**Type country and locality.** Turkey, Brussa [i.e. immediate surroundings of the city of Bursa (Mann 1864: 173)].

**Distribution in Turkey.** Bursa (as *Cinygma tibiale*; Ulmer 1920); Bursa (Tshernova and Belov 1982); Bolu, Erzurum, Hakkari (Kazancı and Braasch 1988); listed from Turkey: Tanatmış (1999); Kazancı (2001b); Kazancı and Türkmen (2012).

**Note.** Endemic to Turkey.

**Comment.** Generic placement is not clear, placed in genus *Epeiron* Demoulin, 1964 by Kluge (2004). The ‘paratype‘ in Museum Hamburg discussed by Tshernova and Belov (1982) represents in fact a syntype, two other syntypes in Natural History Museum Vienna. Larva is not known. The collector Josef Johann Mann (1804-1889) worked since 1837 in the k. k. Zoologisches Hofkabinett in Vienna.

***Rhithrogena theischingeri* Braasch, 1981**

**Type country and locality.** Turkey, Van Gölü (the type locality is the located in the district of the Tatvan, the province of Van) (Braasch 1981).

**Distribution in Turkey.** Van (Braasch 1981); listed from Turkey: Tanatmış (1999); Kazancı (2001b); Kazancı and Türkmen (2012).

**Note.** Endemic to Turkey.

**Comment.** Larva not described.

***Rhithrogena zelinkai* Sowa & Soldán, 1984**

**Distribution in Turkey.** Giresun, Rize, Trabzon (Türkmen and Kazancı 2015); listed from Turkey: Eastern Black Sea Basin (Türkmen and Kazancı, 2013).

**Comment.** So far considered to represent a central Palaearctic taxon, distribution in Turkey is probably questionable (Bauernfeind and Soldán 2012: 378). Imago was not described, for a detailed redescription of larva see Kłonowska-Olejnik and Godunko (2003).

***Rhithrogena znojkoi* (Tshernova, 1938) [sub Ecdyonurus ? znojkoi**]

**Distribution in Turkey.** Erzurum (as *Epeiron znojkoi*; Braasch 1983b); Ardahan, Bayburt, Hakkari (as *Epeiron znojkoi*; Kazancı 1985a); Hatay (as *Rhithrogena znojkoi*; Koch 1988); Antalya, Ankara, Artvin, Bingöl, Erzincan, İçel, Kahramanmaraş, Tunceli (as *Rhithrogena znojkoi*; Kazancı and Braasch 1988); Erzincan, Hakkari, Şırnak (Kazancı 2009); listed from Turkey: Kazancı (2001b); Kazancı and Türkmen (2012).

**Comment.** Not to be confused with *Iron znojkoi* Tshernova, 1938 (presently placed in *Epeorus*). For the generic placement and redescriptions see Thomas and Dia (1982: 297) and Sartori and Sowa (1992: 32).

#### Genus *Heptagenia* Walsh, 1862

##### Subgenus Dacnogenia Kluge, 1988

**Heptagenia (Dacnogenia) coerulans Rostock, 1878**

**Distribution in Turkey.** Ankara (Kazancı 1986a); Şanlıurfa (Koch 1988); Balıkesir (Tanatmış 2000); Erzincan, Erzurum (Kazancı 2001a); Aydın, Çankırı, Yozgat (Kazancı 2001b); Balıkesir, Bursa (Tanatmış 2002); Balıkesir (Tanatmış 2005); Hakkari (Kazancı 2009); listed from Turkey: Tanatmış (1999); Kazancı and Türkmen (2012).

**Comment.** *Dacnogenia* Kluge, 1988 (originally proposed as a subgenus of *Heptagenia*) is considered of generic rank by various authors.

**Heptagenia (Dacnogenia) coerulans micracantha Kluge, 1989**

**Distribution in Turkey.** Zonguldak (as *Heptagenia coerulans*; Tanatmış 2004a); Karabük, Zonguldak (Tanatmış 2005); listed from Turkey: Kazancı and Türkmen (2012).

##### Subgenus Heptagenia Walsh, 1862

**Heptagenia (Heptagenia) longicauda (Stephens, 1836)**

**Distribution in Turkey.** Eskişehir (Kazancı 1986a); Balıkesir (Tanatmış 2000); Ankara (Kazancı 2001b); Balıkesir, Bursa, Kütahya (Tanatmış 2002); Bolu, Karabük, Zonguldak (Tanatmış 2004a); Çanakkale (Narin and Tanatmış 2004); Sinop (Ertorun and Tanatmış 2004); listed from Turkey: Tanatmış (1999); Kazancı and Türkmen (2012).

**Heptagenia (Heptagenia) perflava Brodsky, 1930**

**Distribution in Turkey.** Siirt (Kazancı 2009); listed from Turkey: Kazancı and Türkmen (2012).

**Comment.** Sometimes placed in the genus *Sigmoneuria* Demoulin, 1964, considered to represent a junior subjectiv synonym of *Sigmoneuria amseli* Demoulin, 1964 (see Kluge 1997b: 176). Sometimes confused with *Heptagenia samochai* Demoulin, 1973.

**Heptagenia (Heptagenia) sulphurea (O.F. Müller, 1776)**

**Distribution in Turkey.** Eskişehir (Tanatmış 1995); Ankara, Muğla (Kazancı 2001b); listed from Turkey: Tanatmış (1999), Kazancı and Türkmen (2012).

#### Genus *Thalerosphyrus* Eaton, 1881

***Thalerosphyrus determinatus* (Walker, 1853)**

**Distribution in Turkey.** Ankara (as *Thalerosphyrus* (?); Demoulin 1965); Elazığ (Berker 1981); listed from Turkey: Kazancı (2001b), Kazancı and Türkmen (2012).

**Comment.** Usually considered to represent an exclusively Oriental taxon, occurrence in Turkey is rather unlikely. Association of imaginal stages are somewhat doubtful, for a redescription of larvae see Sartori (2014). A careful re-evaluation of Turkish records based on a re-examination of voucher specimens seems necessary.

### Family LEPTOPHLEBIIDAE Banks, 1900

#### Genus *Calliarcys* Eaton, 1881

***Calliarcys van* Godunko & Bauernfeind in Godunko, Sroka, Soldán & Bojková, 2015**

**Type country and locality.** Turkey, Bitlis Province, Kavuşşahap Dağları mountain range, Pınarca Çayı [river] and its small unnamed right tributary above Kuşlu village, 38°22'32"N, 42°15'31"E, 1720 m a.s.l., about 20 km S of Tatvan town (western shore of the Van Lake) (Godunko et al. 2015).

**Distribution in Turkey.** Bitlis, İzmir (Godunko et al. 2015).

**Note.** Endemic to Turkey.

#### Genus *Choroterpes* Eaton, 1881

##### Subgenus Choroterpes Eaton, 1881

**Comment.** Frequently considered of generic rank (Kluge 2004; Barber-James et al. 2013).

**Choroterpes (Choroterpes) picteti Eaton, 1871**

**Distribution in Turkey.** Ankara, Bingöl (Kazancı 1985a); Diyarbakır (Koch 1985); İstanbul (Tanatmış 1997); Balıkesir, Kütahya (Tanatmış 2000); Balıkesir, Bursa (Tanatmış 2002); Zonguldak (Tanatmış 2004a); Kastamonu, Sinop (Tanatmış 2004b); Balıkesir, Çanakkale (Narin and Tanatmış 2004); Sinop (Ertorun and Tanatmış 2004); Bartın (Tanatmış and Ertorun 2006); Düzce, Zonguldak (Tanatmış 2007); Ankara (Kazancı and Girgin 2008); listed from Turkey: Tanatmış (1999); Kazancı (2001b); Kazancı and Türkmen (2012).

##### Subgenus Euthraulus Barnard, 1932

**Comment.** Frequently considered of generic rank (Kluge 2004; Barber-James et al. 2013).

**Choroterpes (Euthraulus) balcanica (Ikonomov, 1961)**

**Distribution in Turkey.** Trakya (Kazancı 2001b); listed from Turkey: Kazancı and Türkmen (2012).

**Comment.** Imago not described.

#### Genus *Paraleptophlebia* Lestage, 1917

**Comment.** Sometimes considered to represent a subgenus of *Leptophlebia* (see Kluge 1997a).

***Paraleptophlebia submarginata* (Stephens, 1836)**

**Distribution in Turkey.** Erzincan (Kazancı 1986a); Eskişehir (Tanatmış 1995); Kırklareli (Tanatmış 1997); Balıkesir, Kütahya (Tanatmış 2000); Erzurum (Kazancı 2001a); Eskişehir, Muğla (Kazancı 2001b); Kütahya (Tanatmış 2002); Bolu, Kastamonu (Tanatmış 2004a); Kastamonu (Tanatmış 2004b); Bolu (Kazancı and Türkmen 2008a); Bolu (Kazancı and Türkmen 2008b); listed from Turkey: Tanatmış (1999); Kazancı and Türkmen (2012).

***Paraleptophlebia cincta* (Retzius, 1783)**

**Material.** İzmir, Kamberler, male subimago, female subimago, 21.5.1992, H. Malicky leg., 38°21'N, / 27°36'E (NMW).

**Distribution in Turkey.** Bolu (Kazancı and Türkmen 2008a); Bolu (Kazancı and Türkmen 2008b); listed from Turkey: Kazancı and Türkmen (2012).

***Paraleptophlebia werneri* Ulmer, 1920**

**Distribution in Turkey.** Edirne, İstanbul, Kırklareli, Tekirdağ (Tanatmış 1997); Eskişehir (Kazancı 2001b); Zonguldak (Tanatmış 2004a); Kastamonu (Tanatmış 2004b); Sinop (Ertorun and Tanatmış 2004); Sinop (Tanatmış and Ertorun 2008); Bolu (Kazancı and Türkmen 2008a); Bolu (Kazancı and Türkmen 2008b); Kütahya (Aydınlı and Ertorun 2015); listed from Turkey: Tanatmış (1999); Kazancı and Türkmen (2012).

#### Genus *Habroleptoides* Schoenemund, 1929

***Habroleptoides caucasica* Tshernova, 1931**

**Distribution in Turkey.** Bolu (Kazancı 2001b); listed from Turkey: Kazancı and Türkmen (2012).

**Comment.** Larvae is rather similar to *Habroleptoides pontica* Kluge, 1994 and other related taxa.

***Habroleptoides confusa* Sartori & Jacob, 1986**

**Distribution in Turkey.** Bolu, Çankırı (as *Habroleptoides modesta* Hagen, 1864; Kazancı 1985a); Tekirdağ (Tanatmış 1997); Balıkesir (Tanatmış 2000); Kütahya (as *Habroleptoides confuse*; Tanatmış 2002); Bolu, Kastamonu (Tanatmış 2004a); Kastamonu, Sinop (Tanatmış 2004b); Bartın (Tanatmış and Ertorun 2006); Zonguldak (Tanatmış 2007); Sinop (Tanatmış and Ertorun 2008); Konya (Özyurt and Tanatmış 2011); Giresun, Rize, Trabzon (as *Habroleptoides confuse*; Türkmen and Kazancı 2015); listed from Turkey: Tanatmış (1999); Kazancı (2001b); Kazancı and Türkmen (2012); Eastern Black Sea Basin (Türkmen and Kazancı 2013).

**Comment.** Prior to the paper by Sartori and Jacob (1986) authors had confused *Habroleptoides modesta* Hagen, 1864 [endemic to Corsica and Sardinia] with central European taxa.

***Habroleptoides kavron* Kazancı & Türkmen, 2011**

**Type country and locality.** Turkey The stream that is inflowing Büyük Deniz Lake (the type locality is located in the Kaçkar Mountains, Upper Kavron Highland, the province of Rize) (Kazancı and Türkmen 2011).

**Distribution in Turkey.** Rize (Kazancı and Türkmen 2011); listed from Turkey: Kazancı and Türkmen (2012).

**Note.** Endemic to Turkey.

**Comment.** Larva not described. Imagines very similar to *Habroleptoides confusa* Sartori and Jacob, 1986 and other related taxa, hardly separable without doubt.

***Habroleptoides umbratilis* Eaton, 1884**

**Distribution in Turkey.** Bursa, Eskişehir, Kütahya (Tanatmış 1995); listed from Turkey: (Tanatmış 1999); Kazancı and Türkmen (2012).

**Comment.** Larva is similar to *Habroleptoides confusa* and related taxa; discriminating characters provided by Biancheri (1957) most probably insufficient for reliable separation.

#### Genus *Habrophlebia* Eaton, 1881

***Habrophlebia fusca* (Curtis, 1834)**

**Distribution in Turkey.** Antalya, İçel (Puthz 1972); Artvin, Elazığ (Kazancı 1985a); İstanbul, Kırklareli (Tanatmış 1997); Artvin (Kazancı 2001a); listed from Turkey: Tanatmış (1999); Kazancı (2001b); Kazancı and Türkmen (2012).

**Comment.** Occurrence in Turkey is rather questionable, probably based on misidentification or confused with mediterranean *Habrophlebia eldae* Jacob and Sartori, 1984.

***Habrophlebia lauta* Eaton, 1884**

**Distribution in Turkey.** Ankara, Bolu, Giresun, Trabzon (Kazancı 1985a); Bursa, Eskişehir (Tanatmış 1995); İstanbul, Kırklareli, Tekirdağ (Tanatmış 1997); Kütahya (Tanatmış 2000); Çankırı (Kazancı 2001b); Bursa, Kütahya (Tanatmış 2002); Bolu, Kastamonu, Karabük, Zonguldak (Tanatmış 2004a); Kastamonu, Sinop (Tanatmış 2004b); Balıkesir (Narin and Tanatmış 2004); Sinop (Ertorun and Tanatmış 2004); Bartın (Tanatmış and Ertorun 2006); Düzce, Zonguldak (Tanatmış 2007); Bolu (Kazancı and Türkmen 2008a); Bolu (Kazancı and Türkmen 2008b); Sinop (Tanatmış and Ertorun 2008); Balıkesir (Türkmen and Özkan 2011); Afyon (Özyurt and Tanatmış 2011); Kütahya, Manisa (Aydınlı and Ertorun 2015); listed from Turkey: Tanatmış (1999); Kazancı and Türkmen (2012).

#### Genus *Thraulus* Eaton, 1881

***Thraulus bellus* Eaton, 1881**

**Distribution in Turkey.** Sinop (Tanatmış 2004b); listed from Turkey: Kazancı and Türkmen (2012).

**Comment.** Occurrence in Turkey is rather questionable, probably based on misidentification (confusion with *Thraulus thraker*).

***Thraulus thraker* Jacob, 1988**

**First record from Turkey. Material.** Yalova, rivulet I Köseler, Koruköy, ♀SI, 38°50'N, 27°10'E, 200 m a.s.l., 30.5.1992, H. Malicky leg. (NMW).

**Comment.** Larva is not described. Imagines are rather similar to *Thraulus bellus* Eaton, but easily separable by colouration of extreme wing roots (sooty black) and egg chorionic structures (figured in Bauernfeind and Soldán 2012).

### Family EPHEMERIDAE Latreille, 1810

#### Genus *Ephemera* Linnaeus, 1758

***Ephemera danica* Müller, 1764**

**Distribution in Turkey.** Bolu (Kazancı 1984); Bursa, Eskişehir, Kütahya (Tanatmış 1995); İstanbul, Kırklareli (Tanatmış 1997); Kütahya (Tanatmış 2000); Ankara, Balıkesir, Bolu (Kazancı 2001b); Kütahya (Tanatmış 2002); Bolu (Tanatmış 2004a); Kastamonu (Tanatmış 2004b); Zonguldak (Tanatmış 2007); Ankara (Kazancı and Girgin 2008); Bolu (Kazancı and Türkmen 2008a); Bolu (Kazancı and Türkmen 2008b); Sinop (Tanatmış and Ertorun 2008); Balıkesir (Türkmen and Özkan 2011); Afyon, Konya (Özyurt and Tanatmış 2011); Giresun (Türkmen and Kazancı 2015); listed from Turkey: Tanatmış (1999); Kazancı and Türkmen (2012); Eastern Black Sea Basin (Türkmen and Kazancı 2013).

***Ephemera glaucops* Pictet, 1843**

**Distribution in Turkey.** Eskişehir (Kazancı 2001b); listed from Turkey: Kazancı and Türkmen (2012).

**Comment.** Frequently placed in subgenus Sinephemera Kluge, 2004 which is especially well characterized in male imagines (shape of titillator).

***Ephemera lineata* Eaton, 1870**

**Distribution in Turkey.** Bolu (Kazancı 2001b); listed from Turkey: Kazancı and Türkmen (2012).

***Ephemera romantzovi* Kluge, 1988**

**First record from Turkey. Material.** İzmir, Kozak, W Anatolia, 39°17'N, 26°59'E, 250 m a.s.l., female imago, 31.5.1992, Malicky and Sipahiler leg., (NMW).

***Ephemera vulgata* Linnaeus, 1758**

**Distribution in Turkey.** Muş (Braasch 1981); Bolu, Eskişehir (Kazancı 1984); Bolu, Bursa, Eskişehir, Kütahya (Tanatmış 1995); Balıkesir (Tanatmış 2000); Erzurum, Kars (Kazancı 2001a); Bolu, Denizli, Eskişehir (Kazancı 2001b); Bolu, Karabük, Zonguldak (Tanatmış 2004a); Kastamonu, Sinop (Tanatmış 2004b); Sinop (Ertorun and Tanatmış, 2004); Bartın (Tanatmış and Ertorun 2006); Düzce, Zonguldak (Tanatmış 2007); Bolu (Kazancı and Türkmen 2008a); Bolu (Kazancı and Türkmen 2008b); Hakkari, Kars (Kazancı 2009); Malatya (Aydınlı 2013); listed from Turkey: Tanatmış (1999); Kazancı and Türkmen (2012).

***Ephemera zettana* Kimmins, 1937**

**Distribution in Turkey.** Kütahya (Kazancı 1986a); listed from Turkey: Tanatmış (1999); Kazancı (2001b); Kazancı and Türkmen (2012).

### Family PALINGENIIDAE Albarda in Selys-Longchamps, 1888

#### Genus *Palingenia* Burmeister, 1839

***Palingenia anatolica* Jacob, 1977**

**Type country and locality.** Turkey, immediate vicinity of Silifke, Göksu River (the type locality is located in the district of Silifke, the province of İçel) (Jacob 1977).

**Distribution in Turkey.** İçel (Jacob 1977); listed from Turkey: Tanatmış (1999); Kazancı (2001b); Kazancı and Türkmen (2012).

**Note.** Endemic to Turkey.

**Comment.** Larva is not described.

### Family POLYMITARCYIDAE Banks, 1900

#### Genus *Ephoron* Williamson, 1802

***Ephoron virgo* (Olivier, 1791)**

**Distribution in Turkey.** Bingöl (Kazancı 1984); Balıkesir, Bursa (Tanatmış 2000); Erzurum (Kazancı 2001a); Ankara (Kazancı 2001b); Balıkesir (Tanatmış 2002); Bolu, Karabük, Zonguldak (Tanatmış 2004a); Çanakkale (Narin and Tanatmış 2004); Sakarya (Kazancı 2013); Ardahan (Kazancı and Türkmen 2015); listed from Turkey: Tanatmış (1999); Kazancı and Türkmen (2012).

### Family POTAMANTHIDAE Albarda in Selys-Longchamps, 1888

#### Genus *Potamanthus* Pictet, 1843

***Potamanthus luteus* (Linné, 1767)**

**Distribution in Turkey.** Ankara, Bolu, Çankırı (Kazancı 1984); Ankara, Bolu, Bursa, Eskişehir, Kütahya (Tanatmış 1995); Edirne (Tanatmış 1997); Balıkesir, Bursa (Tanatmış 2000); Erzincan, Erzurum (Kazancı 2001a); Ankara, Aydın, Bolu, Çankırı, Denizli, Muğla (Kazancı 2001b); Balıkesir, Bursa, Kütahya (Tanatmış 2002); Bolu, Karabük, Zonguldak (Tanatmış 2004a); Kastamonu, Sinop (Tanatmış 2004b); Sinop (Ertorun and Tanatmış 2004); Bartın (Tanatmış and Ertorun 2006); Düzce, Zonguldak (Tanatmış 2007); Ankara (Kazancı and Girgin 2008); Bolu (Kazancı and Türkmen 2008a); Bolu (Kazancı and Türkmen 2008b); Sinop (Tanatmış and Ertorun 2008); Erzurum, Kars (Kazancı 2009); Malatya (Aydınlı 2013); Giresun (Türkmen and Kazancı 2015); listed from Turkey: Tanatmış (1999); Kazancı and Türkmen (2012); Eastern Black Sea Basin (Türkmen and Kazancı 2013).

### Family EPHEMERELLIDAE Klapálek, 1909

#### Genus *Ephemerella* Walsh, 1862

***Ephemerella mucronata* (Bengtsson, 1909)**

**Distribution in Turkey.** Sivas (Kazancı 2001b); listed from Turkey: Kazancı and Türkmen (2012).

***Ephemerella notata* Eaton, 1887**

**Distribution in Turkey.** Ankara, Bolu, Muğla (Kazancı 2001b); Tokat (Kazancı et al. 2012); listed from Turkey: Kazancı and Türkmen (2012).

***Ephemerella ignita* (Poda, 1761)**

**Distribution in Turkey.** Antalya, İzmir (Puthz 1972); Bolu (Braasch 1981); Ankara, Ardahan, Bingöl, Bolu, Erzincan, Erzurum, Kars, Muş, Sivas, Tunceli, Van (Kazancı 1984); Şanlıurfa (Koch 1988); Bilecik, Bolu, Bursa, Eskişehir, Kütahya (Tanatmış 1995); Çanakkale, İstanbul, Kırklareli Tekirdağ (Tanatmış 1997); Balıkesir, Kütahya (Tanatmış 2000); Erzincan, Erzurum (Kazancı 2001a); Aydın, Bilecik, Muğla (Kazancı 2001b); Balıkesir, Bursa, Kütahya (Tanatmış 2002); Konya (Kazancı et al. 2003); Bolu, Kastamonu, Karabük, Zonguldak (Tanatmış 2004a); Kastamonu, Sinop (Tanatmış 2004b); Balıkesir, Çanakkale (Narin and Tanatmış 2004); Sinop (Ertorun and Tanatmış 2004); Bartın (Tanatmış and Ertorun 2006); Düzce, Zonguldak (Tanatmış 2007); Ankara (Kazancı and Girgin 2008); Sinop (Tanatmış and Ertorun 2008); Bolu (Kazancı and Türkmen 2008b); Afyon (Özyurt and Tanatmış 2011); Malatya (Aydınlı 2013); Giresun, Rize, (Türkmen and Kazancı 2015); Kütahya, Manisa, Uşak (Aydınlı and Ertorun 2015); listed from Turkey: Tanatmış (1999); Kazancı (2001b); Kazancı and Türkmen (2012); Eastern Black Sea Basin (Türkmen and Kazancı 2013).

**Comment.** Placed in *Serratella* Edmunds, 1959 by some authors (e.g. Jacobus and McCafferty 2008) but generic concept for *Serratella* is in discussion.

***Ephemerella mesoleuca* (Brauer, 1857)**

**Distribution in Turkey.** Karabük (Tanatmış 2004a); listed from Turkey: Kazancı and Türkmen (2012).

**Comment.** Placed in *Teloganopsis* Ulmer, 1939 by Jacobus and McCafferty (2008) and Kluge (2004), but their concepts for *Teloganopsis* differ considerably (e.g., *sensu* Kluge restricted to the Oriental realm).

#### Genus *Drunella* Needham, 1905

***Drunella karia* Kazanci, 199**0

**Type country and locality.** Turkey, Çırpı Köyü (Çırpı Köyü is a village and is located in the province of Muğla (Kazancı 1990a).

**Distribution in Turkey.** Muğla (Kazancı 1990a); Antalya, Muğla (Kazancı 1991); listed from Turkey: Tanatmış (1999); Kazancı (2001b); Kazancı and Türkmen (2012).

**Note.** Endemic to Turkey.

**Comment.** Placed in *Serratella* Edmunds, 1959 by some authors (e.g. Jacobus and McCafferty 2008) but generic concept for *Serratella* in discussion.

***Drunella euphratica* Kazanci, 1987**

**Type country and locality.** Turkey, Yuva Köyü (Yuva Köyü is a village and is located in the district of Kemaliye, the province of Erzincan) (Kazancı 1987b).

**Distribution in Turkey.** Erzincan, Hakkari, Malatya, Tunceli (Kazancı 1987b); Ardahan, Erzincan, Hakkari, Malatya, Tunceli (Kazancı 1991); listed from Turkey: Tanatmış (1999); Kazancı (2001b); Kazancı and Türkmen (2012).

**Note.** Endemic to Turkey.

**Comment.** Placed in *Quatica* Jacobus and McCafferty, 2008 by some authors or in *Torleya* Lestage, 1917 following Kluge (2015). Concept for *Quatica* is still in discussion and probably polyphyletic.

#### Genus *Torleya* Lestage, 1917

***Torleya major* (Klapálek, 1905)**

**Distribution in Turkey.** Ankara, Bolu, Erzincan (Kazancı 1984); Bolu, Kırklareli (Kazancı 2001b); Ankara (Kazancı and Girgin 2008); Bolu (Kazancı and Türkmen 2008a); Bolu (Kazancı and Türkmen 2008b); Ardahan, Erzincan (Kazancı 2009); listed from Turkey: (Tanatmış (1999); Kazancı and Türkmen (2012).

### Family CAENIDAE Newman, 1853

#### Genus *Brachycercus* Curtis, 1834

***Brachycercus harrisellus* Curtis, 1834**

**Distribution in Turkey.** Balıkesir (Tanatmış 2002); listed from Turkey: Kazancı and Türkmen (2012).

#### Genus *Caenis* Stephens, 1835

***Caenis horaria* (Linnaeus, 1758)**

**Distribution in Turkey.** Ankara (Kazancı 2001b); Bolu (Tanatmış 2004a); İzmir (Aydınlı and Ertorun 2015); listed from Turkey: Kazancı and Türkmen (2012).

***Caenis luctuosa* (Burmeister, 1839)**

**Distribution in Turkey.** Ankara, Bolu, Bursa, Eskişehir, Kütahya (Tanatmış 1995); Çanakkale, Edirne, İstanbul, Kırklareli, Tekirdağ (Tanatmış 1997); Muğla (Kazancı 1998a); Balıkesir, Bursa, Kütahya (Tanatmış 2000); Ankara (Kazancı 2001b); Balıkesir, Bursa (Tanatmış 2002); Ankara (Kazancı and Girgin 2008); Bolu (Kazancı and Türkmen 2008a); Bolu (Kazancı and Türkmen 2008b); Giresun (Türkmen and Kazancı 2015); listed from Turkey: Tanatmış (1999); Kazancı and Türkmen (2012); Eastern Black Sea Basin (Türkmen and Kazancı 2013).

***Caenis macrura* Stephens, 1836**

**Distribution in Turkey.** Kocaeli (Verrier 1955); Sivas (Koch 1985); Hatay, Şanlıurfa (Koch 1988); Erzincan, Erzurum (Kazancı 2001a); Ankara, Aydın, Eskişehir, Konya, Muğla (Kazancı 2001b); Balıkesir, Bursa, Kütahya (Tanatmış 2002); Bolu, Karabük, Kastamonu, Zonguldak (Tanatmış 2004a); Kastamonu, Sinop (Tanatmış 2004b); Balıkesir, Çanakkale (Narin and Tanatmış 2004); Sinop (Ertorun and Tanatmış 2004); Bartın (Tanatmış and Ertorun 2006); Düzce, Zonguldak (Tanatmış 2007); Sinop (Tanatmış and Ertorun 2008); Afyon, Konya (Özyurt and Tanatmış 2011); Tokat (Kazancı et al. 2012); Malatya (Aydınlı 2013); Giresun (Türkmen and Kazancı 2015); İzmir, Kütahya, Manisa, Uşak (Aydınlı and Ertorun 2015); listed from Turkey: Tanatmış (1999); Kazancı and Türkmen (2012) Eastern Black Sea Basin (Türkmen and Kazancı 2013).

**Comment.** Discrimination of larvae is frequently difficult. Two subspecies from the Mediterranean have been described by Malzacher (1986).

***Caenis martae* Belfiore, 1984**

**Distribution in Turkey.** Bolu (Kazancı and Türkmen 2008a); Bolu (Kazancı and Türkmen 2008b); Balıkesir (Türkmen and Özkan 2011); Giresun (Türkmen and Kazancı 2015); listed from Turkey: Kazancı and Türkmen (2012); Eastern Black Sea Basin (Türkmen and Kazancı 2013).

**Comment.** Discrimination of larvae is frequently difficult. For micrographs of discriminating characters see Bauernfeind and Lechthaler (2014).

***Caenis pseudorivulorum* Keffermüller, 1960**

**Distribution in Turkey.** Ankara (Kazancı 1986a); Zonguldak (Tanatmış 2007); listed from Turkey: Tanatmış (1999); Kazancı (2001b); Kazancı and Türkmen (2012).

***Caenis rivulorum* Eaton, 1884**

**Distribution in Turkey.** Elazığ (Berker 1981); listed from Turkey: Tanatmış (1999); Kazancı (2001b); Kazancı and Türkmen (2012).

**Comment.** Correct identification of *Caenis* taxa in all stages has been greatly improved by Malzacher (1982, 1984, 1986). Earlier records remain usually doubtful and should be checked.

***Caenis robusta* Eaton, 1884**

**Distribution in Turkey.** Antalya, İzmir (Puthz 1972); Antalya (Malzacher 1986); Bursa (Tanatmış 2002); listed from Turkey: Tanatmış (1999); Kazancı and Türkmen (2012).

### Family PROSOPISTOMATIDAE Laméere, 1917

#### Genus *Prosopistoma* Latreille, 1833

***Prosopistoma orhanelicum* Dalkiran, 2009**

**Type country and locality.** Turkey, Deliballılar site (Deliballılar is located in the district of Orhaneli in Orhaneli stream, the province of Bursa) (Dalkıran 2009).

**Distribution in Turkey.** Bursa (Dalkıran 2009); listed for Turkey: Kazancı and Türkmen (2012).

**Note.** Endemic to Turkey.

**Comment.** Differences between larvae of *Prosopistoma orhanelicum* and *Prosopistoma pennigerum* are, however, rather slight, and discrimination may sometimes be doubtful. Manifestations of morphological characters are age dependent (Dalkıran 2009; Schletterer et al. 2015), imagines of *Prosopistoma orhanelicum* have not been described so far. Discriminating characters for east Palaearctic taxa (larvae) have been summarized by Bojková and Soldán (2015).

***Prosopistoma pennigerum* (O.F. Müller, 1785)**

**Distribution in Turkey.** Diyarbakır (sub *Prosopistoma foliaceum* (Fourcroy, 1785); Koch 1985); listed from Turkey: Tanatmış (1999); Kazancı (2001b); Dalkıran (2009); Kazancı and Türkmen (2012).

**Comment.** Differences between larvae of *Prosopistoma orhanelicum* and *Prosopistoma pennigerum* are, however, rather slight, and discrimination doubtful. Manifestations of morphological characters are age-dependent (Schletterer et al. 2015). Discriminating characters for east Palaearctic taxa (larvae) have been summarized by Bojková and Soldán (2015).

### Ephemeroptera species excluded from the catalogue

***Pseudocloeon inopinum* Gillies 1949**

**Distribution in Turkey.** Elazığ (Berker 1981); listed from Turkey: Tanatmış (1999).

**Comment.** Occurrence of this Oriental taxon in Turkey has most probably been based on misidentified material as already stated by Kazancı (2001b).

***Pseudocloeon rubellum* Navás, 1931**

**Distribution in Turkey.** Elazığ (Berker 1981); listed from Turkey: Tanatmış (1999).

**Comment.** Occurrence of this Oriental taxon in Turkey has most probably been based on misidentified material as already stated by Kazancı (2001b).

***Rhithrogena pellucida* Daggy, 1945**

**Distribution in Turkey.** Elazığ (Berker 1981).

**Comment.** Occurrence of this Nearctic taxon in Turkey has most probably been based on misidentified material as already stated by Kazancı (2001b).

## Results

1. Ephemeroptera fauna of some provinces is remarkable and comparatively well-investigated, whereas some provinces have so far been not or insufficiently studied. Best known provinces are Ankara, Balıkesir Bartın, Bingöl, Bolu, Bursa, Çanakkale, Çankırı, Düzce, Erzincan, Erzurum, Eskişehir, Hakkari, Kars, Kastamonu, Karabük, Kırklareli, Kütahya, Muğla, Muş, Sinop, Tekirdağ, and Zonguldak provinces while Adana, Aksaray, Batman, Burdur, Gaziantep, Karaman, Kilis, Mardin, Niğde, Ordu there was not a single record observed. For faunistic research on Ephemeroptera, priority should be given to Adana, Aksaray, Batman, Burdur, Gaziantep, Karaman, Kilis, Mardin, Niğde, Ordu provinces.
2. Some taxa are known so far only from a single locality or from early records that are in need of updating and a careful re-examination based on modern taxonomic standards. Additionally, it would be advisable to revise discriminating characters for some problematic taxa.
3. Areas which have endemic species and their protection status should eventually be reconsidered due to their expected high endemism ratio.

We hope, however, there will be young scientists who will critically evaluate the present data, confirm or correct taxonomically doubtful records, and complete the missing parts in the taxonomic and faunistic knowledge about Ephemeroptera in Turkey.

### Turkish mayfly diversity

As shown in Table 1, the two most diversified families are the Heptageniidae (62 spp., 39.50% of total), followed by the Baetidae (42 spp., 26.75%). On the other hand, the highest level of endemism (12 spp., 50.00%) is in the Heptageniidae followed by the Oligoneuriidae (3 spp., 12.5%).

**Table 1.** Diversity among the different families occurring in Turkey (Nb = number, % Total = % of total species number, % Fam. = % only within the family).

Family	Diversity		Endemism		
	Nb	% Total	Nb	% Total	% Fam.
Ameletidae	2	1.27	0	0	0
Siphlonuridae	3	1.91	1	4.17	33.33
Baetidae	42	26.75	2	8.33	4.76
Isonychiidae	1	0.64	0	0	0
Oligoneuriidae	7	4.46	3	12.5	42.86
Heptageniidae	62	39.50	12	50	19.35
Leptophlebiidae	14	8.92	2	8.33	14.28
Ephemeridae	6	3.82	0	0	0
Palingeniidae	1	0.64	1	4.17	100
Polymitarcyidae	1	0.64	0	0	0
Potamanthidae	1	0.64	0	0	0
Ephemerellidae	7	4.46	2	8.33	28.57
Caenidae	8	5.10	0	0	0
Prosopistomatidae	2	1.27	1	4.17	50
**Total**	**157**	**100**	**24**	**100**	

As shown in Table 2, the two most diversified genera in Turkey are *Baetis* (34 spp., 21.66%) and *Rhithrogena* (19 spp., 12.10%), followed by *Ecdyonurus* (13 spp., 8.28%). Concerning the level of endemism, *Electrogena* and *Rhithrogena* are also in first position (5 spp., 20.83%), followed by *Oligoneuriella* (3 spp., 12.5%). It has to be considered, however, that taxonomy of many taxa, especially of Heptageniidae, recorded from Turkey is but poorly understood at present.

**Table 2.** Diversity among the different genera occurring in Turkey (Nb = number, % Total = % of total species number, % Genus = % only within the genus).

Genus	Diversity		Endemism		
	Nb	% Total	Nb	% Total	% Genus
*Ameletus*	1	0.64	0	0	0
*Metreletus*	1	0.64	0	0	0
*Siphlonurus*	3	1.91	1	4.17	33.33
*Baetis*	34	21.66	2	8.33	5.88
*Centroptilum*	1	0.64	0	0	0
*Cloeon*	2	1.27	0	0	0
*Procloeon*	4	2.55	0	0	0
*Pseudocentroptiloides*	1	0.64	0	0	0
*Isonychia*	1	0.64	0	0	0
*Oligoneuriella*	7	4.46	3	12.5	42.86
*Ecdyonurus*	13	8.28	1	4.17	7.69
*Electrogena*	12	7.64	5	20.83	41.66
*Afronurus*	2	1.27	1	4.17	50
*Epeorus*	10	6.37	0	0	0
*Rhithrogena*	19	12.10	5	20.83	26.31
*Heptagenia*	5	3.18	0	0	0
*Thalerosphyrus*	1	0.64	0	0	0
*Calliarcys*	1	0.64	1	4.17	100
*Choroterpes*	2	1.27	0	0	0
*Paraleptophlebia*	3	1.91	0	0	0
*Habroleptoides*	4	2.55	1	4.17	25.00
*Habrophlebia*	2	1.27	0	0	0
*Thraulus*	2	1.27	0	0	0
*Ephemera*	6	3.82	0	0	0
*Palingenia*	1	0.64	1	4.17	100
*Ephoron*	1	0.64	0	0	0
*Potamanthus*	1	0.64	0	0	0
*Ephemerella*	4	2.55	0	0	0
*Drunella*	2	1.27	2	8.33	100
*Torleya*	1	0.64	0	0	0
*Brachycercus*	1	0.64	0	0	0
*Caenis*	7	4.46	0	0	0
*Prosopistoma*	2	1.27	1	4.17	50
**Total**	**157**	**100**	**24**	**100**	

### Endemic species

The endemic species are separated into two groups, those having a wide distribution in Anatolia (macro-endemic species) and those only occurring in a small mountainous massif or in a narrow part of the Taurus or Pontus (micro-endemic species). There are seven macro-endemic species: *Ecdyonurus bimaculatus*, *Electrogena anatolica*, *Electrogena hakkarica*, *Rhithrogena anatolica*, *Rhithrogena tibialis*, *Calliarcys van*, and *Drunella euphratica* and 17 micro-endemic species: *Siphlonurus muchei*, *Baetis elazigi*, *Baetis macrospinosus*, *Oligoneuriella magna*, *Oligoneuriella paulopilosa*, *Oligoneuriella pectinata*, *Electrogena boluensis*, *Electrogena dirmil*, *Electrogena necatii*, *Afronurus madli*, *Rhithrogena pontica*, *Rhithrogena sublineata*, *Rhithrogena theischingeri*, *Habroleptoides kavron*, *Palingenia anatolica*, *Drunella karia*, and *Prosopistoma orhanelicum*.

## Footnotes

1 Prof. Dr. Nilgün Kazancı, Hacettepe University, Turkey

2 Dr. Mustafa Tanatmış, Anadolu University, Turkey

## References

[B1] Aydınlı C (2013) Sultansuyu Çayı’ nın (Malatya) Ephemeroptera (Insecta) Limnofaunası. Anadolu University Journal of Science and Technology-C 3: 9–14.

[B2] Aydınlı C Ertorun N (2015) Species records of Ephemeroptera (Insecta) nymphs in the Gediz River basin with a new record for the Turkish fauna: *Labiobaetis atrebatinus* Eaton, 1870. Turkish Journal of Zoology 39: 587–595. doi: 10.3906/zoo-1402-64

[B3] Barber-James H Sartori M Gattolliat J-L Webb J (2013) World checklist of freshwater Ephemeroptera species. World Wide Web electronic publication Available from: http://fada.biodiversity.be/group/show/35 [cited 2015 Sep 15]

[B4] Bauernfeind E Haybach A (2012) Case 3594. *Ecdyonurus* Eaton, 1868 and *Ephemera venosa* Fabricius, 1775 (currently *Ecdyonurus venosus*; Insecta, Ephemeroptera): proposed conservation of usage by designation of a neotype for *Ephemera venosa*. The Bulletin of Zoological Nomenclature 69: 1–6.

[B5] Bauernfeind E Lechthaler W (2014) Ephemeroptera – Key to Larvae from Central Europe. EUTAXA, CD-Edition, Vienna, Austria, 1 CD-ROM.

[B6] Bauernfeind E Soldán T (2012) The Mayflies of Europe (Ephemeroptera). Apollo Books, Leiden, 781 pp.

[B7] Belfiore C Tanatmış M Kazancı N (2000) Taxonomy of *Electrogena antalyensis* (Kazancı and Braasch) (Ephemeroptera, Heptageniidae). Aquatic Insects 22: 261–270. doi: 10.1076/0165-0424(200010)22:4;1-Y;FT261

[B8] Berker F (1981) Keban Barajı ve Keban’a dökülen nehirler ile Elazığ bölgesinin Ephemeroptera (Insecta) Limnofaunasının (Larvalarının) Saptanması ve Sistematik İncelenmesi. Fırat University Medical Journal of Health Sciences 6: 124–139.

[B9] Biancheri E (1957) Note sugli Efemerotteri italiani. VII. Descrizione della ninfa e dell’immagine femmina di Habrophlebia (Habroleptoides) umbratilis Eaton. Bollettino Della Società Entomologica Italiana 87: 157–160.

[B10] Bojková J Soldán T (2015) Two new species of the genus *Prosopistoma* (Ephemeroptera: Prosopistomatidae) from Iraq and Algeria. Zootaxa 4018: 109–123. doi: 10.11646/zootaxa.4018.1.6 26624031 10.11646/zootaxa.4018.1.6

[B11] Braasch D (1978) *Epeorus znojkoi* und *Iron magnus* sp.n. (Heptageniidae Ephemeroptera) aus dem Kaukasus. Entomologische Nachrichten 22: 65–70.

[B12] Braasch D (1981) Eintagsfliegen aus Anatolien und Iran (Ephemeroptera, Insecta). Faunistische Abhandlungen Staatliches Museum für Tierkunde in Dresden 8: 75–79.

[B13] Braasch D (1983a) *Siphlonurus muchei* n. sp. aus Anatolien (Ephemeroptera, Siphlonuridae). Reichenbachia 21: 185–186.

[B14] Braasch D (1983b) *Rhithrogena iranica* n.sp. aus dem Iran (Insecta, Ephemeroptera). Entomologische Nachrichten und Berichte 27: 69–70.

[B15] Braasch D (2006) Kritische Anmerkungen zur Taxonomie einiger Heptageniidae (Ephemeroptera) aus Mittelasien und dem Fernen Osten). Entomologische Nachrichten und Berichte 50: 197–204.

[B16] Dalkıran N (2009) A new species of *Prosopistoma* Latreille, 1833 (Ephemeroptera: Prosopistomatidae) from northwestern Turkey. Aquatic Insects 31: 119–131. doi: 10.1080/01650420802642414

[B17] Demoulin G (1963) Mission E. Janssens en Anatolie (Aout-Septembre 1962) Ephemeroptera. Bulletin de l’Institut Royal des Sciences Naturelles de Belgique Entomologie 39: 1–6.

[B18] Demoulin G (1964) Mission H.G. Amsel en Afghanistan (1956) Ephemeroptera. Bulletin del’Institut Royal des Sciences Naturelles de Belgique Entomologie 100: 351–363.

[B19] Demoulin G (1965) Resultats de l’expédition Belge au Moyen-Orient (Avril-Aout 1963) Ephemeroptera. Bulletin de l’Institut Royal des Sciences Naturelles de Belgique Entomologie 41: 1–8.

[B20] Demoulin G (1973) Contribution à l’étude des Ephéméroptères d`Israël. Introduction et I. Heptageniidae. Bulletin de l’Institut Royal des Sciences Naturelles de Belgique Entomologie 49: 1–19.

[B21] Ertorun N Tanatmış M (2004) Karasu Çayı (Sinop)’ nın Ephemeroptera (Insecta) Limnofaunası. Anadolu Üniversitesi Bilim ve Teknoloji Dergisi 5: 107–114.

[B22] Gattolliat J-L Sartori M (2008) What is *Baetis rhodani* (Pictet, 1843) (Insecta, Ephemeroptera, Baetidae) Designation of a neotype and redescription of the species from its original area. Zootaxa 1957: 69–80.

[B23] Geldiay R (1949) Çubuk ve Emir Gölünün Makro ve Mikro Faunasının Mukayeseli Olarak İncelenmesi. Communications Faculty of Sciences University of Ankara 2: 146–252.

[B24] Godunko RJ Palatov DM Martynov AV (2015) Mayflies of the Caucasus Mountains. III. A new representative of the subgenus Rhodobaetis Jacob, 2003 (Baetidae: Baetis) from the South-Western Caucasus. Zootaxa 3948: 182–202. doi: 10.11646/zootaxa.3948.2.2 25947771 10.11646/zootaxa.3948.2.2

[B25] Godunko RJ Prokopov GA Kluge NJu Novikova EA (2004) Mayflies of the Crimean Peninsula. II. *Baetis braaschi* Zimmermann, 1980 (= *B. stipposus* Kluge, 1982 syn. n.) (Ephemeroptera: Baetidae). Acta Zoologica Cracoviensia 47: 155–166. doi: 10.3409/173491504783995807

[B26] Godunko RJ Sroka P Soldán T Bojková J (2015) The higher phylogeny of Leptophlebiidae (Insecta: Ephemeroptera), with description of a new species of *Calliarcys* Eaton, 1881. Arthropod Systematics & Phylogeny 73: 259–279.

[B27] Godunko RJ Vidinova Y Soldán T (2015) Redescription of Ecdyonurus (Ecdyonurus) russevi Braasch and Soldán, 1985 (Ephemeroptera: Heptageniidae). Zootaxa 3915: 551–568. doi: 10.11646/zootaxa.3915.4.6 25662143 10.11646/zootaxa.3915.4.6

[B28] International Commission on Zoological Nomenclature (2007) Opinion 2171, Case 3322. Bulletin of Zoological Nomenclature 64(2): 131 [*Baetis nexus* Navás, 1918 placed on the Official List of Specific Names in Zoology]

[B29] International Commission on Zoological Nomenclature (2015) Opinion 2356, Case 3594. Bulletin of Zoological Nomenclature 72(2): 162–163. [*Ecdyonurus* Eaton, 1868 and *Ephemera venosa* Fabricius, (currently *Ecdyonurus venosus*; Insecta, Ephemeroptera): usage conserved by designation of a neotype for *Ephemera venosa*]

[B30] Jacob U (1977) *Palingenia anatolica* n. sp. (Ephemeroptera, Palingeniidae) aus der Türkei. Entomologische Nachrichten 21: 177–182.

[B31] Jacob U (1988) *Thraulus thraker* sp. n. aus Bulgarien (Insecta, Ephemeroptera: Leptophlebiidae). Faunistische Abhandlungen. Staatliches Museum für Tierkunde in Dresden 26: 1–3.

[B32] Jacob U Sartori M (1984) Die europäischen Arten der Gattung *Habrophlebia* Eaton (Ephemeroptera: Leptophlebiidae). Entomologische Abhandlungen. Staatliches Museum für Tierkunde in Dresden 48: 45–52.

[B33] Jacobus LM McCafferty WP (2008) Revision of Ephemerellidae genera (Ephemeroptera). Transactions of the American Entomological Society 134: 185–274. doi: 10.3157/0002-8320(2008)134[185:ROEGE]2.0.CO;2

[B34] Kazancı N (1985a) New Ephemeroptera (Insecta) records from Turkey. Aquatic Insects 6: 253–258. [1984, publ. 1985]

[B35] Kazancı N (1985b) *Rhithrogena anatolica* sp.n. (Ephemeroptera: Heptageniidae) from Turkey. Mittheilungen der Schweizer. Entomologischen Gesellschaft 58: 311–313.

[B36] Kazancı N (1986a) New Ephemeroptera records from Turkey. Zoology in the Middle East 1: 141–143. doi: 10.1080/09397140.1986.10637539

[B37] Kazancı N (1986b) A new Ephemeroptera (Heptageniidae) species from Anatolia. Turkish Journal of Biology 10: 391–393.

[B38] Kazancı N (1987a) *Ecdyonurus necatii*, a new Ephemeroptera (Heptageniidae) species from Turkey. Aquatic Insects 9: 17–20. doi: 10.1080/01650428709361264

[B39] Kazancı N (1987b) New *Drunella* (Ephemeroptera, Ephemerellidae) species from Turkey. Mittheilungen der Schweizer Entomologischen Gesellschaft 60: 379–382.

[B40] Kazancı N (1990a) *Drunella karia* n. sp. a second species of the genus *Drunella* (Ephemeroptera: Ephemerellidae) from Turkey. Hydrobiologia 199: 35–42. doi: 10.1007/BF00007832

[B41] Kazancı N (1990b) On Heptageniidae (Insecta: Ephemeroptera) Fauna of Turkey II: Genus *Electrogena* Zurwerra et Tomka, 1985. Hacettepe Bulletin of Natural Sciences and Engineering 2: 169–180.

[B42] Kazancı N (1991) Contribution on the zoogeography of Asia Minor based on the distribution of *Drunella* (Ephemeroptera: Ephemerellidae) species. In: Alba-Tercedor J Sanchez-Ortega A (Eds) Overview and strategies of Ephemeroptera and Plecoptera, Proc. 6th Int. Conf. Ephemeroptera and 10th Int. Symp. Plecoptera; 1989 Jul 24–30; Granada, Spain Sandhill Crane Press, 271–276.

[B43] Kazancı N (1992) On Heptageniidae (Ephemeroptera). Fauna of Turkey I: A new species of the genus *Afronurus* Lestage, 1924. Mittheilungen der Schweizer Entomologischen Gesellschaft 65: 1–4.

[B44] Kazancı N (1998a) Additional Ephemeroptera (lnsecta) Records from Turkey and their Zoogeography. Proceeding of the 6^th^ European Congress of Entomology. 1998 Aug 23–29; Ceske Budejovice, Czech Republic, 418–419.

[B45] Kazancı N (1998b) Burdur Gölü ve Acıgöl’ün Limnolojisi, Çevre Kalitesi ve Biyolojik Çeşitliliği. Türkiye İçsuları Araştırmaları Dizisi III, Ankara.

[B46] Kazancı N (2001a) Gümüşhane, Erzurum, Erzincan, Artvin, Kars İlleri Ephemeroptera Faunası Üzerine Ön Çalışma. Türkiye İç Suları Araştırmaları Dizisi V (Ed. Nilgün Kazancı). İmaj Yayınevi, Ankara.

[B47] Kazancı N (2001b) Türkiye Ephemeroptera (Insecta) Faunası. Türkiye İç Suları. Araştırma Dizisi IX, İmaj Yayınevi, Ankara.

[B48] Kazancı N (2009) Ephemeroptera (Insecta) Fauna of Turkey: Records from Eastern Anatolia (Turkey). Review of Hydrobiology 2: 187–195.

[B49] Kazancı N (2011) Record of *Siphlonurus aestivalis* (Eaton, 1903) (Insecta: Ephemeroptera) swarms within surroundings of Beyşehir Lake (Turkey) and its habitat properties. Review of Hydrobiology 4: 59–61.

[B50] Kazancı N (2013) The swarm of *Ephoron virgo* (Olivier, 1791) (Ephemeroptera: Polymitarcyidae) as nuptial behaviour in Sakarya River (Turkey). Review of Hydrobiology 6: 69–80.

[B51] Kazancı N Braasch D (1986) Zwei neue Heptageniidae (Ephemeroptera) aus Anatolien. Mittheilungen der Schweizer Entomologischen Gesellschaft 59: 365–368.

[B52] Kazancı N Braasch D (1988) On some new Heptageniide (Ephemeroptera) from Anatolia. Faunistische Abhandlungen Staatliches Museum für Tierkunde in Dresden 15: 131–135.

[B53] Kazancı N Girgin S (2008) Ephemeroptera, Odonata, Plecoptera (Insecta) fauna of Ankara Stream (Turkey). Review of Hydrobiology 1: 37–44.

[B54] Kazancı N Thomas AGB (1989) Complements et corrections a la faune des Ephemeropteres du Proche-Orient: 2. *Baetis kars* n. sp. de Turquie. Mittheilungen der Schweizer Entomologischen Gesellschaft 62: 323–327.

[B55] Kazancı N Türkmen G (2008a) Research on Ephemeroptera (Insecta) fauna of Yedigöller National Park (Bolu, Turkey): water quality and reference habitat indicators. Review of Hydrobiology 1: 53–72.

[B56] Kazancı N Türkmen G (2008b) Ephemeroptera (Insecta) Türlerinin Bir Koruma Alanındaki Akarsuların Habitat Özelliklerini ve Koruma Alanı Sınırlarını Belirlemede İndikatör Olarak Kullanılması. Ege Journal of Fisheries and Aquatic Sciences 25: 325–331.

[B57] Kazancı N Türkmen G (2011) *Habroleptoides kavron* sp. n., a new species (Ephemeroptera, Leptophlebiidae) from Eastern Black Sea Region (Turkey) with ecological notes. Review of Hydrobiology 4: 63–72.

[B58] Kazancı N Türkmen G (2012) The checklist of Ephemeroptera (Insecta) species of Turkey. Review of Hydrobiology 5: 143–156.

[B59] Kazancı N Türkmen G (2015) The swarm of *Ephoron virgo* (Olivier, 1791) (Ephemeroptera: Polymitarcyidae) in Kura River (Turkey). Review of Hydrobiology 8: 63–50.

[B60] Kazancı N Gökçe Oğuzkurt D Dügel M (2003) Beyşehir Gölü, limnolojisi, çevre kalitesi, biyolojik çeşitliliği ve koruması. Türkiye İç Suları Araştırması VII İmaj Yayıncılık, Ankara.

[B61] Kazancı N İzbırak A Çağlar S Gökçe D (1992) Köyceğiz-Dalyan Özel Çevre Koruma Bölgesi Sucul Ekosisteminin Hidrobiyolojik Yönden İncelenmesi. Özyurt Matbaası, Ankara.

[B62] Kazancı N Türkmen G Bolat HA (2012) Habitat characteristics of endangered species *Marthamea vitripennis* (Burmeister 1839) (Insecta, Plecoptera). Review of Hydrobiology 5: 1–18.

[B63] Kimmins DE (1960) The Ephemeroptera types of species described by A.E. Eaton, R. McLachlan and F. Walker, with particular reference to those in the British Museum (Natural History). Bulletin of the Natural History Museum Entomology series 9: 269–318.

[B64] Kłonowska-Olejnik M Godunko RJ (2003) Contribution to the taxonomy of the Central European species of *Rhithrogena loyolaea* species-group (Ephemeroptera: Heptageniidae). In: Gaino E (Ed.) Research Update on Ephemeroptera and Plecoptera. Università di Perugia, Italy Proceedings of the 2001 International Joint Meeting, X International Conference on Ephemeroptera, XIV International Symposium on Plecoptera held August 5-11, 2001 in Perugia, Università di Perugia, Italy, 2003.

[B65] Kłonowska-Olejnik M (2004) Redescription of *Electrogena quadrilineata* (Landa, 1969) from type material (Ephemeroptera, Heptageniidae). Aquatic Insects 26: 85–95. doi: 10.1080/01650420412331325828

[B66] Kluge NJ (1988) Reviziya rodov sem. Heptageniidae (Ephemeroptera). 1. Diagnozi trib, rodov i podrodov podsem. Heptageniinae. Entomologicheskoe Obozrenie 67: 291–313.

[B67] Kluge NJ (1997a) New subgenera of Holarctic mayflies (Ephemeroptera: Heptageniidae, Leptophlebiidae, Ephemerellidae). Zoosystematica Rossica 5: 233–235.

[B68] Kluge NJ (1997b) Key to freshwater invertebrates of Russia and adjacent lands. Ephemeroptera (St-Petersburg) 3: 176–220.

[B69] Kluge NJ (2004) The phylogenetic system of Ephemeroptera. Kluwer Academic Publishers. doi: 10.1007/978-94-007-0872-3

[B70] Kluge NJ (2015) Ephemeroptera of the World. Available from: http://www.insecta.bio.spbu.ru/z/Eph-spp/Contents.htm [2015 Mar 30]

[B71] Kluge NJ Novikova EA (1992) Reviziya palearkticheskikh rodov i podrodov podenok podsem. Cloeoninae (Ephemeroptera, Baetidae) s opisaniem novykh vidov iz SSSR. Entomologicheskoe Obozrenie 71: 60–83.

[B72] Kluge NJ Novikova EA (2011) Systematics of the mayfly taxon *Acentrella* (Ephemeroptera, Baetidae), with description of new Asian and African species. Russian Entomological Journal 20: 1–56.

[B73] Koch S (1980) Beschreibung der Larve von *Oligoneuriella orontensis* n. sp. aus dem Vorderen Orient und Vergleich mit den paläarktischen Arten von *Oligoneuriella* Ulmer (Ephemeroptera). Ergebnisse der Reisen von R. Kinzelbach im Vorderen Orient Nr XX. Entomologische Zeitschrift 90: 153–160.

[B74] Koch S (1985) Eintagsfliegen aus der Türkei und Beschreibung einer neuen *Baetis*-Art: *B. macrospinosus* n. sp. (Insecta: Ephemeroptera: Baetidae). Senckenbergiana biologica 66: 105–110.

[B75] Koch S (1988) Mayflies of the northern Levant (Insecta: Ephemeroptera). Zoology in the Middle East 2: 89–112. doi: 10.1080/09397140.1988.10637565

[B76] Malzacher P (1981) Beitrag zur Taxonomie europäischer *Siphlonurus*-Larven (Ephemeroptera, Insecta). Stuttgarter Beitraege zur Naturkunde Serie A 345: 1–11.

[B77] Malzacher P (1982) Eistrukturen europäischer Caenidae (Insecta, Ephemeroptera). Stuttgarter Beitraege zur Naturkunde Serie A 356: 1–15.

[B78] Malzacher P (1984) Die europäischen Arten der Gattung *Caenis* Stephens (Insecta: Ephemeroptera). Stuttgarter Beitraege zur Naturkunde Serie A 373: 1–48.

[B79] Malzacher P (1986) Diagnostik, Verbreitung und Biologie der europaischen *Caenis*-Arten (Ephemeroptera: Caenidae). Stuttgarter Beitraege zur Naturkunde Serie A 387: 1–41.

[B80] Mann J (1864) Nachtrag zur Schmetterling-Fauna von Brussa. Entomologische Monatsschrift 8: 173–190.

[B81] Müller-Liebenau I (1969) Revision der europäischen Arten der Gattung *Baetis* Leach, 1815 (Insecta, Ephemeroptera). Gewässer & Abwasser 48/49: 1–214.

[B82] Narin NO Tanatmış M (2004) Gönen (Balikesir) ve Biga (Çanakkale) Çayları’nın Ephemeroptera (Insecta) Limnofaunası. Balıkesir Üniversitesi Fen Bilimleri Enstitüsü 6: 16–25.

[B83] Novikova EA Kluge KJ (1987) Sistematika roda *Baetis* (Ephemeroptera, Baetidae) s opisaniem novogo vida iz Sredney Asii. Vestnik Zoologii 4: 8–19.

[B84] Özyurt I Tanatmış M (2011) Akşehir (Konya-Afyon) ve Eber (Afyon) gölleri havzalarının Ephemeroptera (Insecta) limnofaunası. Afyon Kocatepe University Journal of Sciences and Engineering 8: 29–39.

[B85] Peru N Thomas A (2003) Compléments et corrections à la faune des Éphéméroptères d’Afrique du Nord. 7. Description complémentaire de *Baetis berberus* Thomas, 1986 [Ephemeroptera, Baetidae]. Ephemera 2001 3: 75–82.

[B86] Puthz V (1972) Einige Ephemeropteren (Insecta) aus der Türkei gesammelt von W. Wittmer (Basel). Mittheilungen der Schweizer Entomologischen Gesellschaft 45: 35–36.

[B87] Puthz V (1973) Ephemeropteren aus den östlichen Mittelmeerländern. Fragmenta Entomologica 9: 15–19.

[B88] Puthz V (1978) Limnofauna Europaea (2nd ed). Gustav Fischer, Stuttgart, 256–263.

[B89] Righetti B Thomas A (2001) *Baetis catharus* Thomas, 1986: description des imagos, comparativement aux espèces ouest-euroméditerranéenes du group *alpinus* Pictet [Ephemeroptera, Baetidae]. Ephemera 2: 73–78.

[B90] Sartori M (1992) Mayflies of Israel (Insecta, Ephemeroptera) I: Heptageniidae, Ephemerellidae, Leptophlebiidae, Palingeniidae. Revue suisse de Zoologie 99: 835–858. doi: 10.5962/bhl.part.79856

[B91] Sartori M (2014) The species of *Thalerosphyrus* Eaton, 1881 (Insecta, Ephemeroptera, Heptageniidae, Ecdyonurinae) in Java and Sumatra, with some *Comments* on the diversity of the genus in the Oriental Realm. ZooKeys 420: 19–39. doi: 10.3897/zookeys.420.7904 10.3897/zookeys.420.7904 PMC4109478 25061369

[B92] Sartori M Jacob U (1986) Revision taxonomique du genre *Habroleptoides* Schoenemund, 1929 (Ephemeroptera, Leptophlebiidae) II. A propos du statut de *Habroleptoides modesta* (Hagen, 1864). Revue Suisse de Zoologie 93: 683–691. doi: 10.5962/bhl.part.79506

[B93] Sartori M Sowa R (1992) New data on some *Rhithrogena* species from the Near- and Middle East (Ephemeroptera; Heptageniidae). Aquatic Insects 14: 31–40. doi: 10.1080/01650429209361458

[B94] Savolainen E (2009) *Baetis jaervii* sp. n. (Ephemeroptera: Baetidae) from northern Europe. Acta Entomologica Fennica 20: 182–185.

[B95] Savolainen E Drotz KM Hoffsten PO Saura A (2007) The *Baetis vernus* group (Ephemeroptera, Baetidae) of northernmost Europe: an evidently, diverse but poorly understood group of mayflies. Acta Entomologica Fennica 18: 160–167.

[B96] Schletterer M Bauernfeind E Lechthaler W (2015) Larval redescription of *Prosopistoma pennigerum* (Müller, 1785) from the River Volga near Rzhev, Tver Region, Russia (Insecta: Ephemeroptera). Proceedings of the Joint Meeting of the XIV International Conference on Ephemeroptera and the XVIII International Symposium on Plecoptera in Aberdeen [submitted].

[B97] Soldán T Landa V (1977) Three new species of the genus *Oligoneuriella* (Ephemeroptera, Oligoneuriidae). Acta Entomologica Bohemoslovaca 74: 10–15.

[B98] Sowa R Soldán T Kazancı N (1986) *Rhithrogena pontica* sp.n. (Ephemeroptera: Heptageniidae) from Turkey. Aquatic Insects 8: 67–69. doi: 10.1080/01650428609361232

[B99] Sroka P Bojková J Soldán T Godunko RJ (2015) New species of the genus *Oligoneuriella* Ulmer, 1924 (Ephemeroptera: Oligoneuriidae) from Turkey. Zootaxa 4012: 329–350. doi: 10.11646/zootaxa.4012.2.4 26623858 10.11646/zootaxa.4012.2.4

[B100] Sziráki G (2005) A *Baetis pentaphlebodes* Uhelyi, 1966 (Ephemeroptera, Baetidae). Állattani Közlemények 90: 29–32.

[B101] Tanatmış M (1995) Sakarya Nehir Sistemi Ephemeroptera Limnofaunası’nın belirlenmesi üzerine araştırmalar. Türkiye Entomoloji Dergisi 19: 287–298.

[B102] Tanatmış M (1997) On the Ephemeroptera Fauna (Insecta) of Thrace. Zoology in the Middle East 15: 95–106. doi: 10.1080/09397140.1997.10637744

[B103] Tanatmış M (1999) Genel ve Türkiye Zoocoğrafyası. Meteksan, Ankara Türkiye Ephemeroptera türleri ve yayılışları, 739–747.

[B104] Tanatmış M (2000) Susurluk (Simav) Çayı ve Manyas Gölü Havzası’nın Ephemeroptera (Insecta) Faunası. Türkiye Entomoloji Dergisi 24: 55–67.

[B105] Tanatmış M (2002) The Ephemeroptera (Insecta) fauna of Lake Ulubat basin. Turkish Journal of Zoology 26: 53–61.

[B106] Tanatmış M (2004a) Filyos (Yenice) Irmağı Havzası’nın Ephemeroptera (Insecta) faunası. Türkiye Entomoloji Dergisi 28: 229–240.

[B107] Tanatmış M (2004b) Gökırmak Nehir Havzası (Kastamonu) ile Cide (Kastamnonu) - Ayancık (Sinop) arası sahil bölgesinin Ephemeroptera (Insecta) faunası. Türkiye Entomoloji Dergisi 28: 45–56.

[B108] Tanatmış M (2005) Türkiye Ephemeroptera faunası için iki yeni alttür: Heptagenia (Dacnogenia) coerulans micracantha Kluge, 1989 ve Heptagenia (Dacnogenia) coerulans coerulans Rostok, 1977 (Ephemeroptera: Heptageniidae). Türkiye Entomoloji Dergisi 29: 289–294.

[B109] Tanatmış M (2007) Efteni (Melen) Gölü Havzası İle Melenağzı (Düzce) – Zonguldak Arası Sahil Bölgesinin Ephemeroptera (Insecta) Faunası. Anadolu University Journal of Science and Technology 8: 111–119.

[B110] Tanatmış M Ertorun N (2006) Bartın Çayı (Bartın) Havzası’nın Ephemeroptera (Insecta) Limnofaunası. Ege Journal of Fisheries and Aquatic Sciences 23: 145–148.

[B111] Tanatmış M Ertorun N (2008) Kabalı Çayı (Sinop) Havzası’nın Ephemeroptera (Insecta) Limnofaunası. Turkish Journal of Fisheries and Aquatic Sciences 2: 329–331.

[B112] Tanatmış M Haybach A (2010) *Ecdyonurus bimaculatus* n. sp., a new species of mayfly from Turkey (Ephemeroptera, Heptageniidae, Ecdyonurinae). Lauterbornia 69: 131–140.

[B113] Taşdemir A Ustaoğlu M Balık S Sarı HM (2008) Batı Karadeniz Bölgesindeki (Türkiye) Bazı Göllerin Diptera ve Ephemeroptera Faunası. Journal of Fisheries Sciences 2: 252–260.

[B114] Thomas AGB Dia A (1982) *Ecdyonurus* (?) *znojkoi* Tshernova 1938: redescription et appartenance générique réelle (Ephemeroptera, Heptageniidae). Bulletin de la Société d’Histoire Naturelle de Toulouse 118: 297–303.

[B115] Thomas A Marie V Brulin M (1999) Corrections à la faune des Éphémères d’Europe occidentale: 2. *Epeorus assimilis* Eaton, 1885 est une espèce valide, distincte d’*E. sylvicolus* (Pictet, 1865) [Ephemeroptera, Heptageniidae]. Ephemera 1: 85–91.

[B116] Topkara TE Taşdemir A Yıldız S Ustaoğlu RE Balık S (2009) Toros dağ silsilesi üzerindeki bazı göllerin sucul böcek (Insecta) faunasına katkılar. Turkish Journal of Fisheries and Aquatic Sciences 3: 10–17.

[B117] Tshernova OA (1931) Beiträge zur Kenntnis der paläarktischen Ephemeropteren. I Zoologischer Anzeiger 92: 214–218, 1930 [publ. 1931].

[B118] Tshernova OA Belov VV (1982) Systematic position and synonymy of *Cinygma tibiale* Ulmer, 1920 (Ephemeroptera, Heptageniidae). Entomologische Mitteilungen aus dem Zoologischen Museum Hamburg 7: 193–194.

[B119] Türkmen G Kazancı N (2013) The key to the Ephemeroptera (Insecta) larvae in running waters of the Eastern Black Sea Basin (Turkey) with the new records. Review of Hydrobiology 6: 31–55.

[B120] Türkmen G Kazancı N (2015) Additional records of Ephemeroptera (Insecta) species from the Eastern Part of Black Sea Region (Turkey). Review of Hydrobiology 8: 33–50.

[B121] Türkmen G Özkan N (2011) Larval Ephemeroptera records from Marmara Island and Kapıdağ Peninsula (North-Western Turkey) with new record of *Baetis milani* Godunko, Prokopov and Soldán 2004. Review of Hydrobiology 4: 99–113.

[B122] Ulmer G (1920) Neue Ephemeropteren. Archiv für Naturgeschichte Abteilung A 85: 1–80. [publ. 1920]

[B123] Verrier ML (1955) Éphéméroptères capturés en Turquie et en Iran par M. K. Lindberg. Bulletin de la Societe Entomologique de France 60: 98.

[B124] Webb JM McCafferty WP (2008) Heptageniidae of the World. Part II: Key to the Genera. Canadian Journal of Arthropod Identification 7: 1–55.

[B125] Yanai Z Sartori M Dor R Dorchin N (2016) Molecular phylogeny and morphological analysis resolve long-standing controversy over generic concepts in the Ecdyonurinae (Ephemeroptera: Heptageniidae). Systematic Entomology 41. [in print]

